# Haloboration of Internal Alkynes with Boronium and Borenium Cations as a Route to Tetrasubstituted Alkenes**

**DOI:** 10.1002/anie.201302609

**Published:** 2013-06-06

**Authors:** James R Lawson, Ewan R Clark, Ian A Cade, Sophia A Solomon, Michael J Ingleson

**Affiliations:** School of Chemistry, University of ManchesterManchester, M13 9PL (UK) E-mail: Michael.ingleson@manchester.ac.uk

**Keywords:** borenium ions, boronate esters, haloboration, synthetic methods, tetrasubstituted alkenes

Vinyl boronates are extremely useful precursors, especially for the formation of new C–C bonds by cross-coupling and conjugate addition reactions.^1^, ^2^ Although alkyne hydroboration is a powerful synthetic route,^3^, ^4^ it is not applicable to the synthesis of trisubstituted vinyl boronates. Thus, alternative regio- and stereospecific methods are needed, particularly for subsequent use in the formation of tetrasubstituted alkenes,^5^–^7^ as the production of these important biologically active compounds as single isomers by classical methods is challenging.^8^ One simple approach to trisubstituted vinyl boronates is the functionalization of internal alkynes by metal-catalyzed 1,2-carboboration^9^–^13^ and 1,1-carboboration.^14^ The introduction of two selectively transformable moieties onto an internal alkyne should enable ready access to tetrasubstituted alkenes by successive cross-coupling reactions. Significant progress has been made in this area, particularly in the dimetalation of internal alkynes to provide two nucleophilic sites of distinct reactivity.^15^–^19^ The haloboration of internal alkynes is an attractive alternative to dimetalation, as it generates ambivalent synthetic intermediates that contain both a nucleophilic and an electrophilic position.^20^ These synthetic intermediates are ideally suited for the diversity-oriented synthesis of tetrasubstituted alkenes. To date, the application of alkyne haloboration with boron trihalides (BX_3_) has been limited to terminal alkynes, and has proved an effective route to produce trisubstituted alkenes with excellent regio- and stereoselectivity.^21^–^25^ The haloboration of internal alkynes is unsuccessful with BCl_3_, and it is either slow^25^ or produces isomeric mixtures susceptible to B–C bond cleavage when BBr_3_ is used.^21^, ^26^ Recent calculations found that the haloboration of internal alkynes with BCl_3_ is endothermic, but as the Lewis acidity of BX_3_ increases (Cl<Br<I), haloboration becomes exothermic, and the energy of the key transition state is also reduced.^27^ This result suggested that an increase in the electrophilicity at boron beyond that of BX_3_ would facilitate the haloboration of internal alkynes.

In the boron analogue of the Friedel–Crafts reaction, three-coordinate [X_2_BL]^+^ borocations (termed borenium cations; X=halide, L=amine)^28^, ^29^ were considerably stronger electrophiles towards arene nucleophiles than BX_3_.^30^, ^31^ Borenium cations were thus expected to be highly reactive towards other π nucleophiles, and a recent report on borenium-ion-catalyzed alkene hydroboration supports this premise.^32^ However, when an alkyne and [X_2_BL]^+^ are combined, a range of outcomes are possible beyond the desired alkyne haloboration. By analogy to the reactivity of frustrated Lewis pairs (FLPs),^33^ both dehydroboration and Lewis base addition are also feasible (Scheme 1). We envisaged that systems in which the Lewis base coordinates strongly to boron throughout the reaction would favor the haloboration of alkynes, as continuous base coordination precludes the presence of a free base, which is essential for both dehydroboration and Lewis base addition.^30^ Herein we report that the borocation-based haloboration of internal alkynes is indeed possible and proceeds with excellent regio- and stereoselectivity. A reaction sequence consisting of successive haloboration, esterification, and cross-coupling is demonstrated as an effective route for the construction of analogues of important tetrasubstituted-alkene drug molecules in isomerically pure form.

**Scheme 1:**
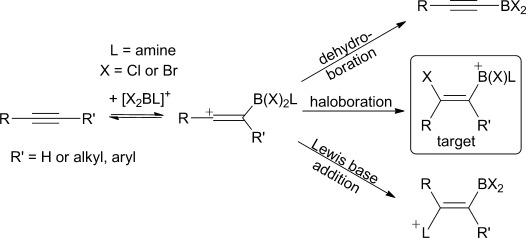
Possible outcomes of the combination of an alkyne with [X_2_BL]^+^.

We chose 2-(*N*,*N*-dimethylamino)pyridine (2-DMAP) as an ideal amine, as it is inexpensive, strongly nucleophilic, and robust to C–N cleavage reactions.^34^ [X_2_B(2-DMAP)][EX_4_] (X=Cl, E=Al: **1**; X=Br, E=B: **2**) and [Ph(Cl)B(2-DMAP)][AlCl_4_] (**3**) were readily synthesized by the sequential addition of 2-DMAP and AlCl_3_ (or BBr_3_) to BX_3_ or PhBCl_2_. In solution (^11^B NMR resonances *δ*_B_=12.1 and 16.4 ppm for **1** and **3**, respectively; **2** is insoluble in noncoordinating halogenated solvents) and in the solid state (the structures of **1** and **3** are shown in Figure 1), the boron center in these compounds is four-coordinate, and 2-DMAP chelation to boron is observed. Coordinative saturation at boron suggested that **1**–**3** may not be viable boron Lewis acids. However, the boracycles in **1** and **3** are significantly strained and contain long Me_2_N–B bond distances (1.697(4) and 1.726(2) Å, respectively) relative to those in most boronium cations (N–B ca. 1.60 Å).^28^, ^29^ The Me_2_N–B distances in **1** and **3** are comparable to those in the strained boronium cation [*N*,*N*′-(9-BBN)-1,8-bis(dimethylamino)naphthalene]^+^ (B–N 1.72–1.73 Å; 9-BBN=9-borabicyclo[3.3.1]nonane),^35^ which does react as a boron Lewis acid at 20 °C.

**Figure 1:**
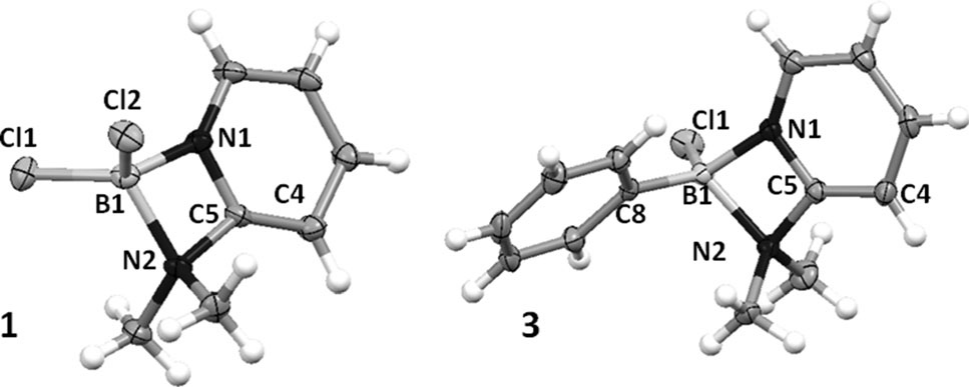
ORTEP representations of the cationic portions of **1** and **3** with ellipsoids drawn at the 50 % probability level. Selected bond distances [Å] and angles [°] for **1**: B1–N1 1.567(4), B1–N2 1.697(4); N2-C5-N1 99.1(2), C4-C5-N2 136.5(3); for **3**: B1–N1 1.588(2), B1–N2 1.726(2); N2-C5-N1 100.37(14), C4-C5-N2 135.27(15).

A low barrier to ring opening was predicted for **1** by calculations at the M06-2X/6-311G(d,p) (PCM, CH_2_Cl_2_) level. Experimental confirmation of facile ring opening was forthcoming from the rapid reaction of **1** with PPh_3_ at 20 °C to form the boronium cation [Cl_2_B(PPh_3_)(2-DMAP)][AlCl_4_], in which 2-DMAP is now a monodentate ligand. Thus, **1** can be viewed as a masked form of a borenium cation that is an intramolecular FLP, termed **1_RO_**. The ring-opened form of **1**, **1_RO_**, was calculated to be 12.3 kcal mol^−1^ higher in energy than four-coordinate **1**; thus, a relatively minor modification of the structure to increase the electron density on boron was expected to favor the open-ring isomer. Indeed, [(*o*-catecholato)B(2-DMAP)][AlCl_4_] (**4**), in which the chloride ligands have been replaced by aryloxy ligands with higher π basicity, does exist in the ring-opened form in solution and in the solid state (Figure 2). Compound **4** has structural dimensions very similar to those calculated for **1_RO_**. In particular, the bond distances are consistent with a significant degree of Me_2_N=C and B=N multiple-bond character.

**Figure 2:**
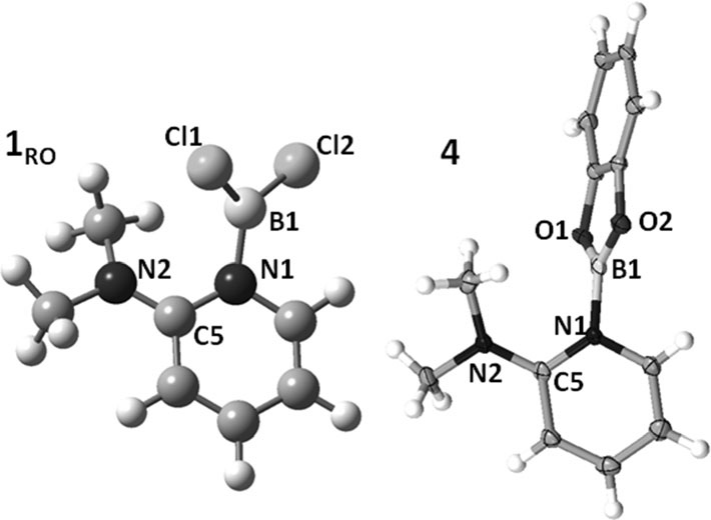
Left: Calculated structure for **1_RO_**. Right: ORTEP representation of the cationic portion of **4** with thermal ellipsoids drawn at the 50 % probability level. Selected bond distances [Å] for **1_RO_**: N1–B1 1.49, N2–C5 1.33; for **4**: N1–B1 1.472(5), N2–C5 1.340(4).

Studies on the reactivity of **1** towards alkynes started with the more reactive terminal alkynes as substrates. Haloboration, dehydroboration, and Lewis base addition were all feasible. The addition of *tert*-butylacetylene (1 equiv) to **1** resulted in the formation of a single product containing a vinylic C–H moiety and a four-coordinate boron center (as determined by ^1^H and ^11^B NMR spectroscopy). Confirmation of *syn* haloboration and the expected regiochemical outcome on the basis of electronic and steric factors was provided by single-crystal X-ray diffraction analysis of the product derived from the haloboration of *tert*-butylacetylene, [*cis*-Cl(*t*Bu)C=C(H)(BCl(2-DMAP))][AlCl_4_] (**5**; Scheme 2). Compound **5** is stable in solution: after 6 days in dichloromethane, no other haloboration isomers were observed. Compound **1** could be used for the haloboration of two equivalents of a terminal alkyne, and the addition of a further equivalent of *tert*-butylacetylene to **5** also gave the double-haloboration product, [(*cis*-Cl(*t*Bu)C=C(H))_2_B(2-DMAP)][AlCl_4_]. The reaction of **3** with alkynes is potentially more complex, since both haloboration and carboboration are feasible through Cl^−^ or Ph^−^ migration, respectively.^21^, ^36^ In this case, the haloboration of terminal alkynes with **3** occurred exclusively; no carboboration was observed. Compound **3** reacts with only one equivalent of a terminal alkyne, thus precluding a subsequent carboboration step upon the addition of more of the alkyne. The reactivity of **1** and **3** stands in contrast to that of FLPs containing neutral borane Lewis acids. Such FLPs undergo addition of the Lewis base/Lewis acid across the alkyne or dehydroboration.^33^, ^37^ As the chelating catechol moiety in **4** precludes anion migration, compound **4** does display FLP-type reactivity. With **4**, exclusive dehydroborylation of alkynes was observed (72 h at 60 °C; Scheme 2, left), with no Lewis base addition products formed.^38^

**Scheme 2:**
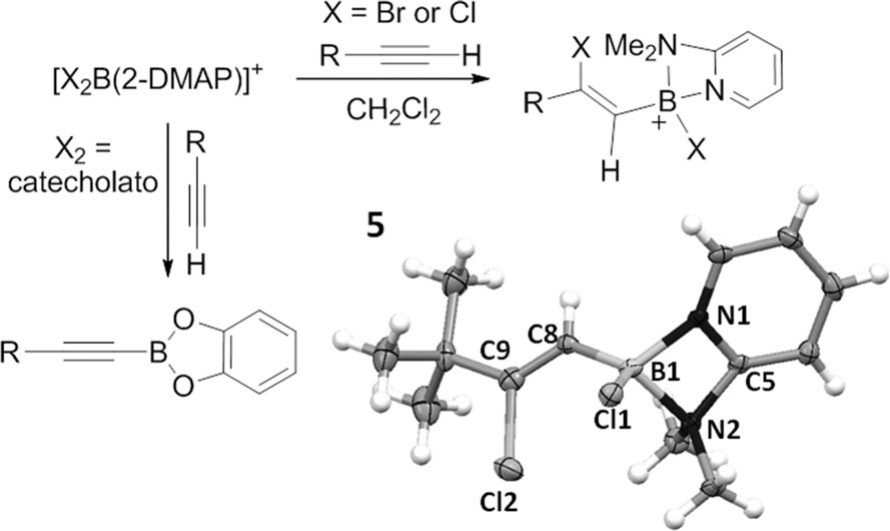
Haloboration and dehydroboration of terminal alkynes, as determined by the nature of the ligand X. Bottom right: ORTEP representation of the cationic portion of **5** with ellipsoids drawn at the 50 % probability level.

To probe the scope of the haloboration, we treated a range of terminal alkynes with **1**. In each case, a single *syn*-haloboration isomer was produced (Table 1, entries 1–5). Subsequent esterification to give the corresponding pinacol boronate esters proceeded in good yield with no loss in stereo-/regioisomeric purity. Attempts to expand the reactivity of **1** to the haloboration of internal alkynes were unsuccessful. However, the ring-opened form of the bromine analogue, **2_RO_**, was expected to be more electrophilic than **1_RO_** and concomitantly more reactive towards alkynes as a result of diminished X→B π bonding. Compound **2** was effective for the haloboration of terminal alkynes (e.g., 1-pentyne; Table 1, entry 6) and also internal dialkyl-substituted alkynes (Table 1, entries 7 and 8). Again, esterification provided the vinyl pinacol boronate esters in good yield. The haloboration of an unsymmetrical dialkyl alkyne (Table 1, entry 8) proceeded with excellent regioselectivity to form a single isomer as a result of synergic steric and electronic control. However, less nucleophilic alkynes, including diaryl and aryl/alkyl-substituted alkynes, did not react with **2**.

**Table 1:** Haloboration of alkynes and subsequent esterification

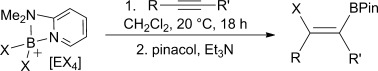				

Entry	X/E	R	R′	Yield [%]^[a,b]^
1	Cl/Al	*t*Bu	H	63
2	Cl/Al	Ph	H	88
3	Cl/Al	Pr	H	73
4	Cl/Al	4-BrC_6_H_4_	H	68
5	Cl/Al	4-MeC_6_H_4_	H	65
6	Br/B	Pr	H	78
7	Br/B	Et	Et	62
8	Br/B	Me	*i*Pr	52

[a] Yield of the isolated product. [b] The isomeric purity was above 99 % in all cases; the alkene geometry was determined by ^1^H NMR spectroscopy through NOE measurements. Pin=2,3-dimethyl-2,3-butanedioxy.

The significant B=N_pyridyl_ character observed in **4** and calculated in the ring-opened borenium-cation forms of **1** and **2** (Figure 2) attenuates the electrophilicity at boron and thus limits the scope of the reaction with respect to the alkyne substrate. The replacement of 2-DMAP with a less nucleophilic amine can be expected to enhance the electrophilicity at boron and thus broaden the scope of the reaction, provided that: 1) halide migration is faster than amine dissociation and subsequent deprotonation or Lewis base addition, and 2) 2-DMAP is not mechanistically crucial. Conceivably, 2-DMAP is only essential if the pendant NMe_2_ group facilitates the transfer of a halide to a carbon atom of the alkyne by concomitant chelation to boron (as in Scheme 3, left). The mechanism of haloboration with **1** was therefore probed at the M06-2X/6-311G(d,p) (PCM CH_2_Cl_2_) level. Calculations were limited to the cationic component and a reaction pathway involving the direct transfer of a chloride from boron to carbon. Chloride transfer mediated by [AlCl_4_]^−^ (through a six-membered transition state; Scheme 3, right) was discounted, as the observed similarity of the reaction profile for the haloboration of terminal alkynes with [Cl_2_B(2-DMAP)][B(3,5-C_6_H_3_Cl_2_)_4_] to that with **1** indicated anion independence.

**Scheme 3:**
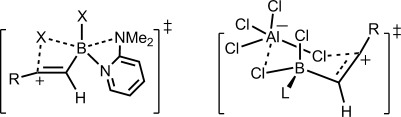
Proposed transition states for concerted and anion-mediated haloboration mechanisms.

The haloboration of alkynes with BX_3_ has been calculated to proceed via a weak van der Waals complex and concerted C–B/C–X bond formation.^27^ In contrast, the sequence of elementary steps calculated for haloboration with [**1**]^+^ proceeds via a strongly bonded vinyl-cation intermediate, **B** (Scheme 4). Intermediate **B** was calculated to contain a B–C bond and two B–Cl bonds with lengths consistent with single bonds to four-coordinate boron (1.66 and 1.83–1.88 Å, respectively). Importantly, the key transition state for haloboration, **TS_BC_**, indicated that the chloride transfer occurs in a separate exothermic step prior to the rechelation of 2-DMAP to boron. These calculations therefore suggest that a pendant base is not essential for chloride transfer; thus, more reactive (than **1_RO_** and **2_RO_**) borenium cations containing less nucleophilic amines were explored for haloboration.

**Scheme 4:**
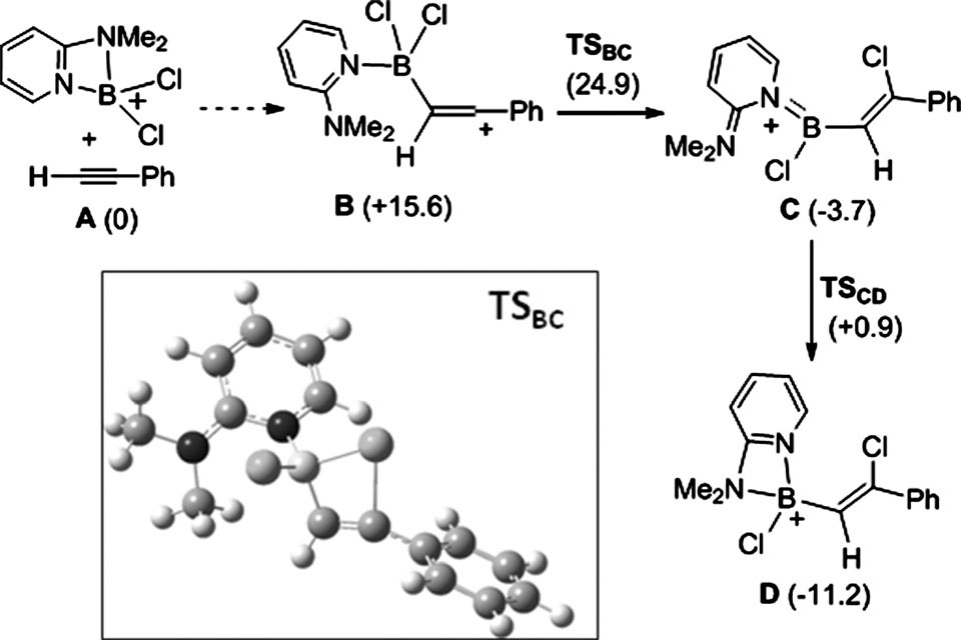
Relative energies (kcal mol^−1^) in CH_2_Cl_2_ of intermediates in the haloboration of phenylacetylene with [**1**]^+^. The structure of the transition state **TS_BC_** is also shown.

The use of our most reactive arene borylating agent, formed from stoichiometric *N*,*N*,4-trimethylaniline (Me_2_NTol), BCl_3_, and AlCl_3_,^39^ only led to intractable mixtures when combined with terminal alkynes. The combination of Me_2_NTol, BCl_3_, and AlCl_3_ produces a complex equilibrium mixture containing both boron and aluminum Lewis acids, and we postulate that the latter lead to undesired reactivity, such as alkyne polymerization. The use of 2,6-lutidine as a bulkier and more nucleophilic amine (as compared to Me_2_NTol) ensures that the equilibrium position lies significantly towards the borenium ion [Cl_2_B(2,6-lutidine)][AlCl_4_] (**6**). Initial reactions between **6** and phenylacetylene confirmed rapid *syn* haloboration and the formation of a single isomer, *cis*-[Cl(Ph)C=C(H)(B(Cl)(2,6-lutidine))]^+^. The lack of evidence for dehydroboration to form PhC≡CBCl_2_ suggests that 2,6-lutidine does not dissociate rapidly from the vinyl-cation intermediate relative to the rate of halide migration. The vinyl chloroborenium cation [Cl(Ph)C=C(H)(B(Cl)(2,6-lutidine))]^+^ is, to the best of our knowledge, the first example of a boron analogue of an allyl cation.

Pleasingly, **6** displayed significantly broader reactivity than that of **1** and **2**. A range of internal alkynes underwent haloboration with **6**, and subsequent in situ esterification provided the trisubstituted vinyl boronate esters with excellent regio- and stereoselectivity. Substrates suitable for haloboration with **6** included dialkyl- (Table 2, entries 2 and 3), alkyl/aryl- (entries 4–6), and diaryl-substituted internal alkynes (entry 7). Isolated products were all derived from *syn* haloboration and are consistent with electronic effects maximizing the stability of the intermediate vinyl cation (e.g., π delocalisation > hyperconjugation). There was also a significant degree of steric control observed: the haloboration of 1-isopropyl-2-methylacetylene with **6** provided only one regioisomer derived from *syn* addition, with the boron atom at the least-hindered position (Table 2, entry 3). The borenium cation **6** can be prepared in situ from BCl_3_/2,6-lutidine/AlCl_3_ without using a glove box (provided 2,6-lutidine is slowly added to BCl_3_, to prevent *ortho*-Me activation);^40^ under these conditions, the yields remained respectable (Table 2, entries 5 and 6). Internal haloalkynes are also amenable to haloboration: 1-bromo-2-phenylacetylene underwent haloboration with **6** to provide a functionality-rich alkene (Table 2, entry 8). 2-Methylhexen-3-yne and allylphenylacetylene both underwent *syn*-1,2-haloboration selectively at the alkyne position to form a single product (Table 2, entries 9 and 10). No haloboration of the alkene moiety was observed, in agreement with the relative energetics calculated for haloboration, according to which alkynes are favored over alkenes.^27^ The addition of **6** to 1-(2-thienyl)acetylene demonstrated that haloboration of the alkyne occurs in preference to direct borylation of the thiophene α position (Table 2, entry 11). Finally, haloboration with **6** tolerates methoxy groups (Table 2, entry 12), but it is not compatible with carbonyl moieties, as previously observed in direct electrophilic arene borylation.

**Table 2:** Haloboration of alkynes with 6

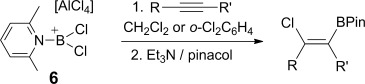				

Entry	R	R′	*t* [h]^[a]^	Yield [%]^[b,c]^
1	Ph	H	4	71
2	Et	Et	24	81
3	*i*Pr	Me	48	83
4	Ph	Me	8	62
5	Ph	Et	24	72
6^[d]^	Ph	Et	18	69
7	Ph	Ph	48	43
8	Ph	Br	48	71
9	Et	C(Me)=CH_2_	48	61
10	Ph	CH_2_CH=CH_2_	4	53
11	2-thienyl	H	48	65
12	4-MeOC_6_H_4_	H	4	52

[a] Reaction time before the addition of Et_3_N/pinacol. [b] Yield of the isolated product. [c] The isomeric purity was above 99 % in all cases; the alkene geometry was determined by ^1^H NMR spectroscopy through NOE measurements. [d] Compound **6** was prepared in situ without using a glove box.

Haloboration with **6** appears to be reversible at 20 °C. The addition of a more nucleophilic internal alkyne led to complete displacement (within 6 h at 20 °C) of the less nucleophilic alkyne in a formal retrohaloboration/haloboration process [Eq. (1)]. The vinyl chloroborenium cation formed by the chloroboration of phenylacetylene with **6** was heated to determine whether retrohaloboration and any subsequent dehydroboration occurred to form an alkynyl dichloroborane. However, no dehydroboration, and more remarkably, minimal isomerization (<5 %), occurred at reflux in dichloromethane (for 3 days). The stability to isomerization of the vinyl chloroborenium cations is remarkable and contrasts starkly with the stereoconversion that occurs during haloboration with BX_3_ (particularly on heating).^26^ Stereoisomerism in alkyne haloboration with BX_3_ was calculated to proceed through a second haloboration of the halo boraalkene, followed by retrohaloboration to give mixtures of *cis*- and *trans*-haloborated alkenes.^27^ In contrast to haloboration with BX_3_, the further haloboration of vinyl chloroborenium cations by **6** will be disfavored owing to Coulombic repulsion between the two cations.


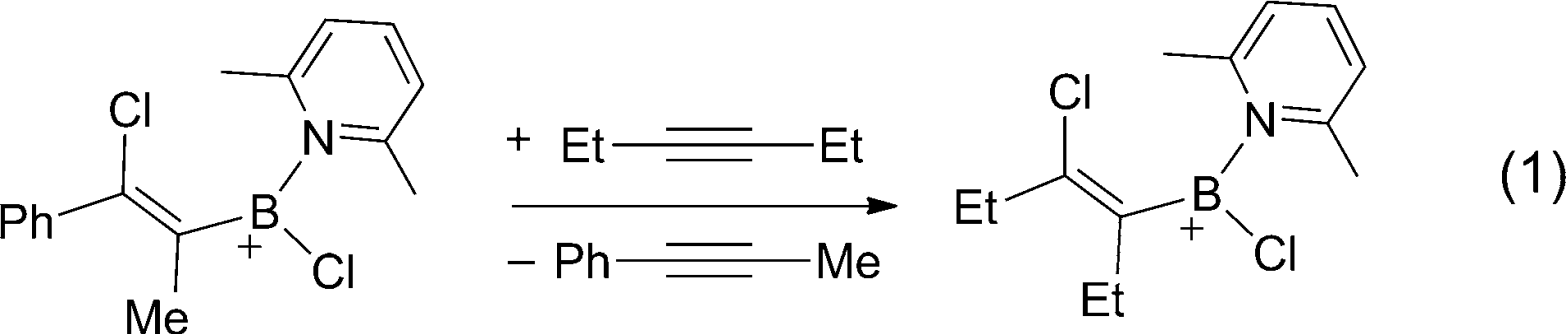
(1)

Vinyl haloboronate esters are potentially versatile precursors for the regio- and stereoselective synthesis of a range of tetrasubstituted alkenes. To confirm their utility, we explored cross-coupling with the vinyl boronate **7**. Pleasingly, Suzuki–Miyaura cross-coupling of **7** with 4-iodotoluene proceeded efficiently in excellent yield to produce only a single isomer of the desired tetrasubstituted alkene (Scheme 5). In this way, an internal alkyne could be converted into a single isomer of a clomifene analogue through a simple and high-yielding two-step route. We also investigated successive Suzuki–Miyaura cross-coupling reactions by first combining **7** with 4-iodotoluene and subsequently coupling the initial product with 4-fluorophenylboronic acid (without the purification of intermediates). Under these nonoptimized conditions, the second cross-coupling step proceeded efficiently, although a minor quantity of a second isomer was produced. The two readily separable isomers of (Ph)(4-FC_6_H_4_)C=C(Ph)(4-MeC_6_H_4_) were produced in a 6:1 ratio, presumably the steric demand of P*t*Bu_3_ induced *cis*–*trans* isomerization during cross-coupling, as previously reported.9b,c

**Scheme 5:**
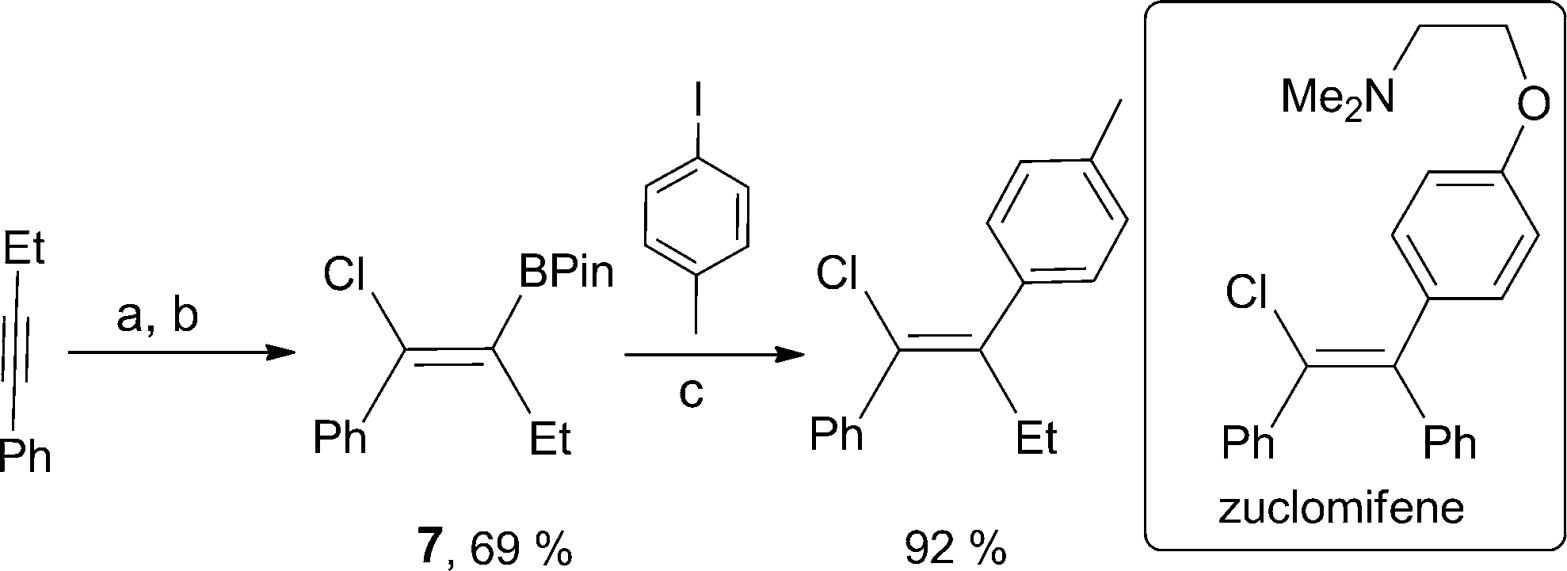
Synthesis of a tetrasubstituted alkene: a) **6**, CH_2_Cl_2_, 18 h; b) Et_3_N, pinacol, c) [Pd_2_(dba)_3_] (5 mol %), P*t*Bu_3_ (20 mol %), KOH (300 mol %), THF/H_2_O. dba=dibenzylideneacetone.

In conclusion, we have synthesized highly strained 2-DMAP-ligated boronium cations that react as functional boron Lewis acids owing to a low barrier to ring opening. The ring-opened isomers possess sufficient electrophilicity for the selective haloboration of terminal alkynes and bromoboration of dialkyl-substituted internal alkynes. More reactive dichloroborenium cations, readily synthesized from inexpensive reagents, enable the chloroboration of a range of internal alkynes. The regio- and stereoselectivity of haloboration is excellent as a result of synergic steric and electronic control and the absence of stereoisomerization. Overall, this first successful haloboration/esterification of internal alkynes is an inexpensive one-pot method for the production of trisubstituted vinyl pinacol boronate esters. These products are useful precursors for the synthesis of biologically active tetrasubstituted alkenes through sequential cross-coupling reactions.

## Experimental Section

Haloboration procedure: Under an inert atmosphere, a solution of 2,6-lutidine (1 equiv) in hexane was added dropwise to a Schlenk tube containing a 1 m solution of boron trichloride in heptanes (1.2 equiv) at 0 °C, whereupon the (2,6-lutidine)BCl_3_ adduct precipitated as a pale yellow-white solid. After 20 min, the solvent was removed under reduced pressure, and (2,6-lutidine)BCl_3_ was suspended in *o*-dichlorobenzene or dichloromethane. Aluminum trichloride was added as a solid to this suspension to generate the borocation **6**, and the resulting mixture was stirred for 20 min. The desired alkyne (1 equiv) was then added dropwise, and the reaction mixture was stirred for 18–48 h (see Table 2). On completion of the haloboration reaction, a solution of pinacol (2.1 equiv) in excess triethylamine was added to the reaction mixture. (**Caution**! This reaction is strongly exothermic.) Volatiles were removed under vacuum, and the product was extracted with pentane. Subsequent filtration through a short plug of silica gel removed impurities and provided the vinyl pinacol boronate ester.

## Supplementary material

As a service to our authors and readers, this journal provides supporting information supplied by the authors. Such materials are peer reviewed and may be re-organized for online delivery, but are not copy-edited or typeset. Technical support issues arising from supporting information (other than missing files) should be addressed to the authors.
