# Supplementary material for: Haloboration of Internal Alkynes with Boronium and Borenium Cations as a Route to Tetrasubstituted Alkenes
Source: Angew Chem Int Ed Engl. 2013 Jun 6;52(29):7518–22. doi: 10.1002/anie.201302609 (PMC3749439; doi:10.1002/anie.201302609)
Supplement: Supplementary file 1 [file anie0052-7518-SD1.pdf]

Angewandte Chemie
Eine Zeitschrift der Gesellschaft Deutscher Chemiker

Supporting Information
© Wiley-VCH 2013
69451 Weinheim, Germany

Haloboration of Internal Alkynes with Boronium and Borenium Cations as a Route to Tetrasubstituted Alkenes**

*James R. Lawson, Ewan R. Clark, Ian A. Cade, Sophia A. Solomon, and Michael J. Ingleson**

anie_201302609_sm_miscellaneous_information.pdf

General Considerations

All manipulations of air and moisture sensitive species were performed under an atmosphere of argon or nitrogen using standard Schlenk and glovebox techniques. Glassware was dried in a hot oven overnight and heated before use. Hexane, *ortho* -dichlorobenzene, d_1-chloroform, d_2-dichloromethane, 2,6-lutidine, Et_3N and were dried over calcium hydride and distilled under vacuum. Pentane and dichloromethane were dried by passing through an alumina drying column incorporated into an MBraun SPS800 solvent purification system. All solvents were degassed and stored over molecular sieves (3Å) under inert atmosphere or in the glovebox. 2,6-lutidineBCl_3 and $[(Cl_2B(2,6\text{-lutidine})][AlCl_4]$ (compound **6**) were made as previously reported.[1] All other materials were purchased from commercial vendors and used as received. NMR spectra were recorded with a Bruker AV-400 spectrometer (400 MHz ^1H; 100 MHz ^{13}C; 128 MHz ^{11}B; 376.50 MHz ^{19}F; 104 MHz ^{27}Al). ^1H NMR chemical shifts are reported in ppm relative to protio impurities in the deuterated solvents and ^{13}C NMR using the solvent resonances unless otherwise stated. ^{11}B NMR spectra were referenced to external $BF_3{:}Et_2O$, and ^{27}Al to $Al(NO_3)_2$ in D_2O $(Al(D_2O)_6{}^{3+})$. Resonances for the carbon directly bonded to boron are not observed in the $^{13}C\{^1H\}$ NMR spectra. Elemental analysis of air sensitive compounds was performed by London Metropolitan University service.

[1] E. R. Clark, A. Del Grosso, M. J. Ingleson, *Chem. Eur. J.*, **2013**, 19, 2462

[BCl_2(2DMAP)][$AlCl_4$] **(1)**

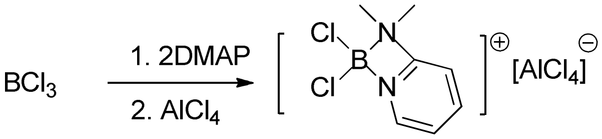

To a 1M solution of BCl_3 (0.83 ml, 6.33 mmol) in anhydrous CH_2Cl_2, 2-dimethylaminopyridine (79 ml, 6.33 mmol) was added. NMR spectroscopy was used to confirm the reaction, to which Aluminium trichloride (0.84 g, 6.33 mmol) was added. A solid precipitated out of solution, and the solvent was removed under reduced pressure, leaving a white solid. This was washed with CH_2Cl_2 (10 ml, x3) and **1** was isolated as a white powder (3.03 g, 8.12 mmol, 81%).

^{1}H NMR (400 MHz, $CDCl_3$): δ 8.88 (td, 1H, ^3J(H,H) =8.2, ^3J(H,H) =1.5), 8.67 (d, 1H, ^3J(H,H) =5.3), 8.43 (d, 1H, ^3J(H,H) =8.6), 8.24 (dd, 1H, ^3J(H,H) =7.8, ^3J(H,H) =5.8), 3.48 (s, 6H, NMe_2) ppm;
^{11}B NMR (128.4 MHz, $CDCl_3$): δ 12.2 (s) ppm.
^{27}Al NMR (104 MHz, $CDCl_3$): δ 103.45 (s) ppm.

Elemental Analysis
Calculated: C 22.71; H 2.72; N 7.57.
Observed: C 22.64; H 2.89; N 7.56.

[BBr_2(2DMAP)][BBr_4] **(2)**

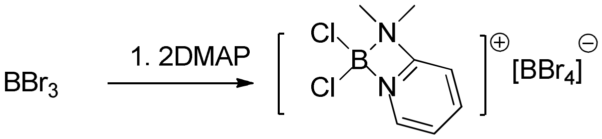

BBr_3 (1.0M solution in heptanes, 0.2 ml, 0.2 mmol) was added to 2DMAP (24 µl, 0.194 mmol) dissolved in DCM (5 ml), producing a colourless precipitate. The solvent was removed *in vacuo* and the resulting power redissolved in DCM (5 ml) and this solution then frozen by immersion in N_2(l). Additional BBr_3 solution (0.2ml as before) was layered upon the frozen reaction mixture, the whole then sealed and allowed to slowly attain room temperature. This slow diffusion-limited reactivity grew crystals of **2** as colourless blocks (60 mg, 0.096 mmol, 51%). The resulting crystals were almost completely insoluble in chlorinated solvents and no NMR data are available.

Elemental Analysis
Calculated: C 13.49; H 1.62; N 4.49.
Observed: C 13.64; H 1.73 ; N 4.61.

[Ph(Cl)B(2DMAP)][$AlCl_4$] **(3)**

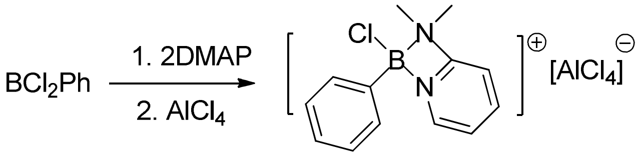

To a 1M solution of BCl_2Ph (0.83 ml, 6.33 mmol) in anhydrous CH_2Cl_2, 2-dimethylaminopyridine (79 ml, 6.33 mmol) was added. NMR spectroscopy was used to confirm the reaction, to which Aluminium trichloride (0.84 g, 6.33 mmol) was added. A solid precipitated out of solution, and the solvent was removed under reduced pressure, leaving a white solid. This was washed with CH_2Cl_2 (10 ml, x3) and **3** was isolated as a white powder (2.02 g, 4.89 mmol, 77%).

^{1}H NMR (400 MHz, CD_2Cl_2): δ 8.84 (td, 1H, ^3J(H,H) =8.2, ^3J(H,H) =1.5), 8.62 (d, 1H, ^3J(H,H) =5.3), 8.32 (d, 1H, ^3J(H,H) =8.6), 8.21 (dd, 1H, ^3J(H,H) =7.8, ^3J(H,H) =5.8), 7.46-7.36 (m, 3H), 7.24 (d, 2H, ^3J(H,H) =6.8), 3.08 (s, 6H, NMe_2) ppm;
^{13}C NMR (100.6 MHz, CD_2Cl_2): δ 155.03, 149.69, 140.59, 131.22, 130.22, 129.24, 127.78, 118.17, 46.88 ppm;
^{11}B NMR (128.4 MHz, CD_2Cl_2): δ 16.4 (s) ppm.
^{27}Al NMR (104 MHz, CD_2Cl_2): δ 103.4 (s) ppm.

Elemental Analysis
Calculated: C 37.68; H 3.65; N 6.76.
Observed: C 37.57; H 3.72; N 6.76.

[CatB(2DMAP)][$AlCl_4$] **(4)**

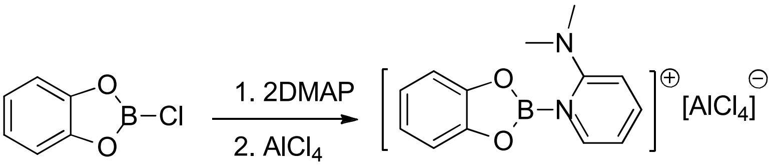

2DMAP (0.13 ml, 1 mmol) was added to a solution of Chlorocatecholborane (154 mg, 1 mmol) in DCM (2 ml) in a greased schlenk, giving immediate formation of a yellow colour. The solvent was removed *in vacuo*, and $AlCl_3$ (133 mg, 1 mmol) was added. The mixture was dissolved in *o*DCB (3 ml) and stirred until dissolution occurred to give a bright yellow solution. The mixture was layered with ^{n}Hexane and crystals grown over 5 days. The crystals were isolated by filtration, washed with *o*DCB (3 ml) and ^{n}Hexane (2x5 ml) and dried in vacuo to give **4** as yellow plates (218 mg, 0.53 mmol, 53%)

^{1}H NMR (400 MHz, CD_2Cl_2): δ 8.15 (d, 1H), 8.02 (t, 1H), 7.45 – 7.43 (, 2H), 7.31 – 7.29 (m, 3H), 7.09 (t, 1H), 3.35 (s, 6H, NMe_2) ppm;
^{13}C NMR (100.6 MHz, CD_2Cl_2): δ 159.38, 147.08, 145.87, 139.10, 125.32, 116.26, 115.18, 114.07, 43.51 ppm;
^{11}B NMR (128.4 MHz, CD_2Cl_2): δ 25.3 (s) ppm.
^{27}Al NMR (104 MHz, CD_2Cl_2): δ 103.4 (s) ppm.
Elemental Analysis
Calculated: C 38.10; H 3.44; N 6.83.
Observed: C 38.14; H 3.44; N 6.75.

Reaction of 1 with PPh_3

A J. Young's fitted NMR tube fitted with a d_6-DMSO capillary was charged with **1** (38mg, 0.1mmol) in DCM ($1cm^3$) and PPh_3 (26mg, 0.1mmol) was added and the NMR recorded immediately. The retained sole sharp peak at 103.22ppm in the ^{27}Al NMR spectrum indicates that the anion $[AlCl_4]$ remains the major ^{27}Al containing species in solution, indicating that halide transfer has not occurred. The ^{11}B NMR spectrum shows some residual **1** (12.1ppm), and two new signals showing B-P coupling. The minor signal at 3.24ppm ($_1J_{B-P}$=155.3Hz) is the simple Cl_3B-PPh_3 adduct, with the major signal at 3.85ppm ($_1J_{B-P}$=153.1Hz) attributed to the boronium species $[(2\text{-DMAP})BCl_2(PPh_3)][AlCl_4]$. After standing overnight, the neutral adduct Cl_3B-PPh_3 is the only B-P containing species in solution. The neutral adduct and borenium signals are found at -1.68ppm and 1.48ppm respectively in the $^{31}P\{^1H\}$ NMR.

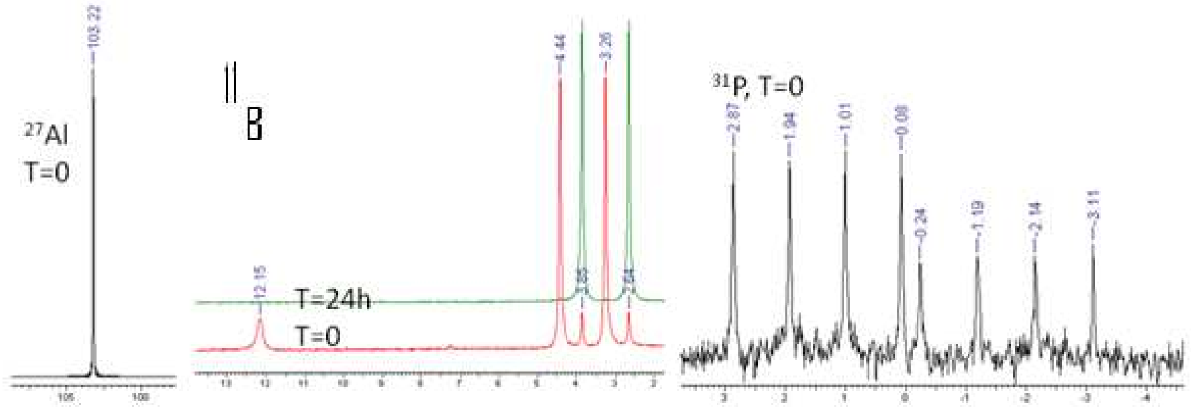

2-(3,3-dimethylbut-1-yn-1-yl)benzo[d][1,3,2]dioxaborole

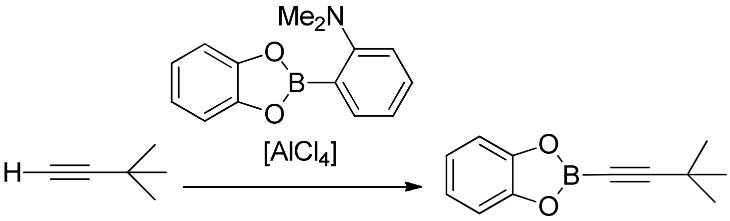

To a solution of [CatB(2DMAP)][$AlCl_4$] (100 mg, 0.24 mmol) in anhydrous CH_2Cl_2 in a J.Young's NMR tube, ^{t}Bu acetylene (30 µl, 0.24 mmol) was added, remaining a clear orange solution. The reaction mixture was then heated at 60˚C for 72 hours, during which time it turned a darker shade orange/brown. NMR spectroscopy was used to confirm reaction completion, and the solution was moved to a Schlenk flask via cannula transfer under argon. The solvent was removed under reduced pressure, leaving a brown oil. Anhydrous pentane was used to extract the product into another Schlenk flask, where it was isolated as a white solid (35 mg, 0.18 mmol, 72%).
^{1}H NMR (400 MHz, CD_2Cl_2): δ 7.14 (dd, 2H, ^3J(H,H) = 8.3), 7.04 (dd, 2H, ^3J(H,H) = 8.3), 1.26 (s, 9H) ppm;
^{13}C NMR (100.6 MHz, CD_2Cl_2): δ147.76, 123.01, 112.51, 30.34, 28.26 ppm;
^{11}B NMR (128.4 MHz, CD_2Cl_2): δ 24.1 ppm.
Elemental Analysis
Calculated: C 72.04; H 6.55.
Observed: C 71.91; H 6.67.

2-(phenylethynyl)benzo[d][1,3,2]dioxaborole

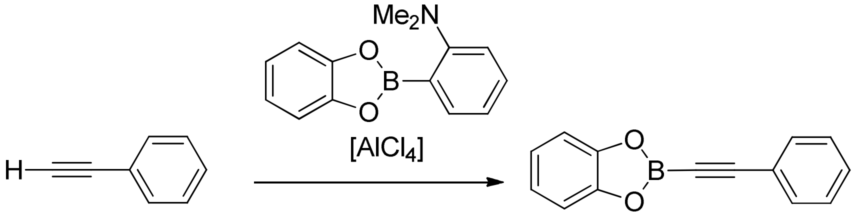

To a solution of [CatB(2DMAP)][$AlCl_4$] (100 mg, 0.24 mmol) in anhydrous CH_2Cl_2 in a J.Young's NMR tube, phenylacetylene (26 µl, 0.24 mmol) was added, remaining a clear orange solution. The reaction mixture was then heated at 60˚C for 72 hours, during which time it turned a darker shade orange/brown. NMR spectroscopy was used to confirm reaction completion, and the solution was moved to a Schlenk flask via cannula transfer under argon. The solvent was removed under reduced pressure, leaving a brown oil. Anhydrous pentane was used to extract the product into another Schlenk flask, where it was isolated as a pale white solid (32 mg, 0.15 mmol, 60%).

^{1}H NMR (400 MHz, CD_2Cl_2): δ 7.55 (d, 2H, ^3J(H,H) = 7.6 Hz), 7.38-7.29 (m, 3H), 7.19 (dd, 2H, ^3J(H,H) = 8.3), 7.08 (dd, 2H, ^3J(H,H) = 8.3) ppm;
^{13}C NMR (100.6 MHz, CD_2Cl_2): δ147.83, 132.79, 130.10, 128.51, 123.22, 121.18, 112.68 ppm;
^{11}B NMR (128.4 MHz, CD_2Cl_2): δ 24.9 ppm.
Elemental Analysis
Calculated: C 76.44; H 4.12.
Observed: C 76.32; H 4.17.

2-(pent-1-yn-1-yl)benzo[d][1,3,2]dioxaborole

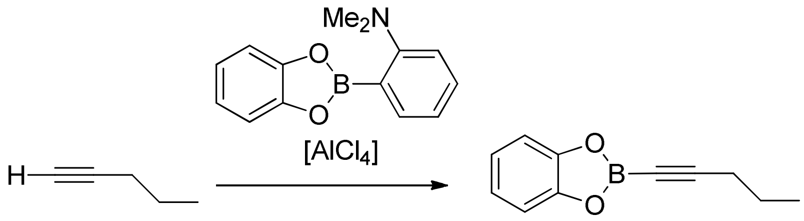

To a solution of [CatB(2DMAP)][AlCl$_4$] (100 mg, 0.24 mmol) in anhydrous CH_2Cl_2 in a J.Young's NMR tube, 1-pentyne (24 µl, 0.24 mmol) was added, remaining a clear orange solution. The reaction mixture was then heated at 60˚C for 72 hours, during which time it turned a darker shade orange/brown. NMR spectroscopy was used to confirm reaction completion, and the solution was moved to a Schlenk flask via cannula transfer under argon. The solvent was removed under reduced pressure, leaving a brown oil. Anhydrous pentane was used to extract the product into another Schlenk flask, where it was isolated as a white solid (31 mg, 0.17 mmol, 69%).

^{1}H NMR (400 MHz, CD_2Cl_2): δ 7.14 (m, 2H, ^3J(H,H) = 8.3), 7.04 (m, 2H, ^3J(H,H) = 8.3) 2.30 (t, 2H, ^3J(H,H) = 7.1 Hz), 1.58 (sextet, 2H, ^3J(H,H) = 7.3 Hz), 0.98 (t, 3H, ^3J(H,H) = 7.4 Hz) ppm;
^{13}C NMR (100.6 MHz, CD_2Cl_2): δ147.76, 123.02, 112.53, 21.99, 13.48 ppm;
^{11}B NMR (128.4 MHz, CD_2Cl_2): δ 24.1ppm.
Elemental Analysis
Calculated: C 71.06; H 5.96.
Observed: C 70.89; H 5.89.

2-((4-ethynylphenyl)ethynyl)benzo[d][1,3,2]dioxaborole

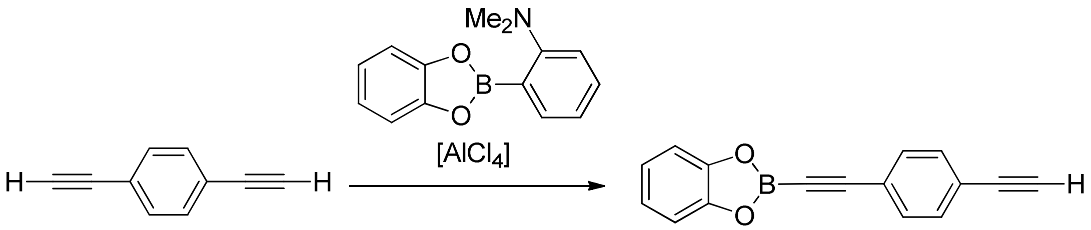

To a solution of [CatB(2DMAP)][AlCl$_4$] (100 mg, 0.24 mmol) in anhydrous CH_2Cl_2 in a J.Young's NMR tube, 1,4-diethynylbenzene (30 mg, 0.24 mmol) was added, remaining a clear orange solution. The reaction mixture was then heated at 60˚C for 72 hours, during which time it turned a darker shade orange. NMR spectroscopy was used to confirm reaction completion, and the solution was moved to a Schlenk flask via cannula transfer under argon. The solvent was removed under reduced pressure, leaving a brown oil. Anhydrous pentane was used to extract the product into another Schlenk flask, where it was isolated as a white powder (44 mg, 0.18 mmol, 74%).

^{1}H NMR (400 MHz, CD_2Cl_2): δ 7.52 (d, 2H, ^3J(H,H) = 8.1 Hz), 7.44 (d, 2H, ^3J(H,H) = 8.1 Hz), 7.21 (dd, 2H), 7.08 (dd, 2H), 3.16 (s, 1H) ppm;
^{13}C NMR (100.6 MHz, CD_2Cl_2): δ 146.75, 131.58, 131.15, 130.99, 122.27, 111.70, 78.92, 78.05 ppm;
^{11}B NMR (128.4 MHz, CD_2Cl_2): δ 24.5 ppm.
Elemental Analysis
Calculated: C 78.76; H 3.72.
Observed: C 78.82; H 3.64.

2-(p-tolylethynyl)benzo[d][1,3,2]dioxaborole

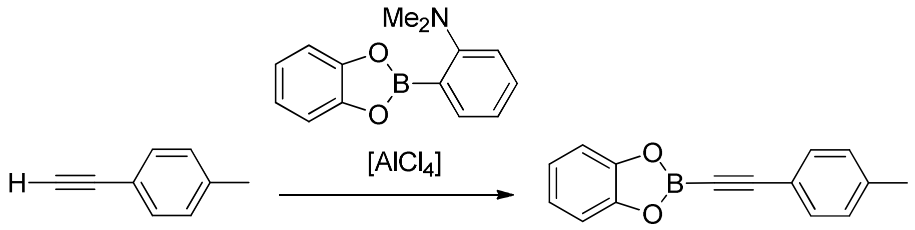

To a solution of [CatB(2DMAP)][$AlCl_4$] (100 mg, 0.24 mmol) in anhydrous CH_2Cl_2 in a J.Young's NMR tube, 4-ethynyltoluene (31 µl, 0.24 mmol) was added, remaining a clear orange solution. The reaction mixture was then heated at 60˚C for 48 hours, during which time it turned a darker shade orange/brown. NMR spectroscopy was used to confirm reaction completion, and the solution was moved to a Schlenk flask via cannula transfer under argon. The solvent was removed under reduced pressure, leaving a brown oil. Anhydrous pentane was used to extract the product into another Schlenk flask, where it was isolated as a white powder (50 mg, 0.21 mmol, 88%).

^{1}H NMR (400 MHz, CD_2Cl_2): δ 7.44 (d, 2H, ^3J(H,H) = 8.1 Hz), 7.20 (dd, 2H, ^3J(H,H) =8.3), 7.12 (d, 2H, ^3J(H,H) = 7.8 Hz), 7.07 (dd, 2H, ^3J(H,H) =8.3), 2.32 (s, 3H, Methyl) ppm;
^{13}C NMR (100.6 MHz, CD_2Cl_2): δ 147.85, 140.63, 132.76, 129.29, 123.16, 118.09, 112.66, 21.72 ppm;
^{11}B NMR (128.4 MHz, CD_2Cl_2): δ 24.8 (s) ppm.
Elemental Analysis
Calculated: C 76.97; H 4.74.
Observed: C 76.93; H 4.73.

2-(3-phenylprop-1-yn-1-yl)benzo[d][1,3,2]dioxaborole

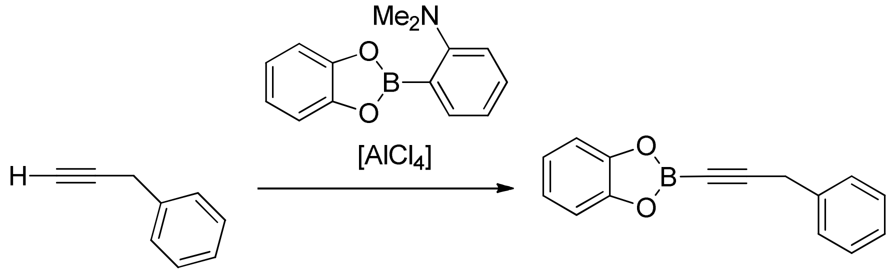

To a solution of [CatB(2DMAP)][$AlCl_4$] (100 mg, 0.24 mmol) in anhydrous CH_2Cl_2 in a J.Young's NMR tube, 3-phenyl-1-propyne (30 µl, 0.24 mmol) was added, remaining a clear orange solution. The reaction mixture was then heated at 60˚C for 24 hours, during which time it turned a darker shade orange. NMR spectroscopy was used to confirm reaction completion, and the solution was moved to a Schlenk flask via cannula transfer under argon. The solvent was removed under reduced pressure, leaving a brown oil. Anhydrous pentane was used to extract the product into another Schlenk flask, where it was isolated as an off-white solid (42 mg, 0.18 mmol, 74%).

^{1}H NMR (400 MHz, CD_2Cl_2): δ 7.34-7.19 (m, 5H), 7.16 (dd, 2H, ^3J(H,H) =8.2), 7.08 (dd, 2H, ^3J(H,H) =8.2), 3.74 (s, 2H) ppm;
^{13}C NMR (100.6 MHz, CD_2Cl_2): δ 147.73, 134.75, 128.76, 128.04, 127.06, 123.13, 112.61, 26.11 ppm;
^{11}B NMR (128.4 MHz, CD_2Cl_2): δ 24.2 (s) ppm.
Elemental Analysis
Calculated: C 77.64; H 3.91.
Observed: C 77.59, H 3.90.

2-(3-chloroprop-1-yn-1-yl)benzo[d][1,3,2]dioxaborole

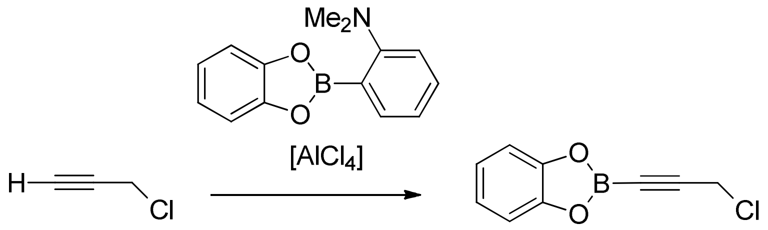

To a solution of [CatB(2DMAP)][$AlCl_4$] (50 mg, 0.12 mmol) in anhydrous CH_2Cl_2 in a J.Young's NMR tube, propargyl chloride (9 µl, 0.12 mmol) was added, remaining a clear orange solution. The reaction mixture was then heated at 60˚C for 72 hours, during which time it turned a darker shade orange/brown. NMR spectroscopy was used to confirm reaction completion, and the solution was moved to a Schlenk flask via cannula transfer under argon. The solvent was removed under reduced pressure, leaving a brown oil. Anhydrous pentane was used to extract the product into another Schlenk flask, where it was isolated as a white powder (17 mg, 0.09 mmol, 77%).

^{1}H NMR (400 MHz, CD_2Cl_2): δ 7.19 (dd, 2H, ^{3}J(H,H) =7.9), 7.07 (dd, 2H, ^{3}J(H,H) =7.9), 4.19 (s, 2H) ppm;
^{13}C NMR (100.6 MHz, CD_2Cl_2): δ 147.56, 123.40, 112.78, 29.78 ppm;
^{11}B NMR (128.4 MHz, CD_2Cl_2): δ 23.5 (s) ppm.
Elemental Analysis
Calculated: C 58.76; H 2.96.
Observed: C 58.69; H 2.90.

2-((4-bromophenyl)ethynyl)benzo[d][1,3,2]dioxaborole

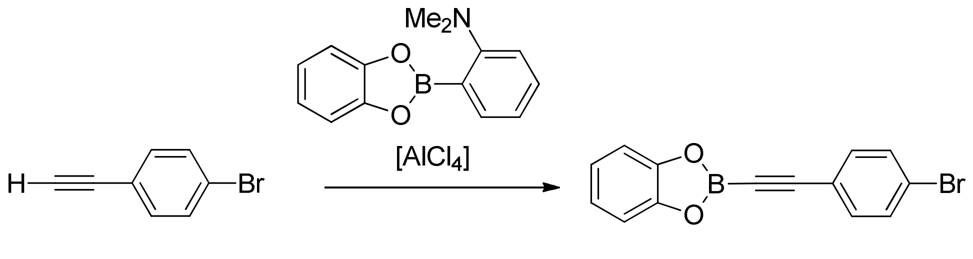

To a solution of [CatB(2DMAP)][$AlCl_4$] (100 mg, 0.24 mmol) in anhydrous CH_2Cl_2 in a J.Young's NMR tube, 4-bromophenylacetylene (44 mg, 0.24 mmol) was added, remaining a clear orange solution. The reaction mixture was then heated at 60˚C for 72 hours, during which time it turned a darker shade orange/brown. NMR spectroscopy was used to confirm reaction completion, and the solution was moved to a Schlenk flask via cannula transfer under argon. The solvent was removed under reduced pressure, leaving a brown oil. Anhydrous pentane was used to extract the product into another Schlenk flask, where it was isolated as a very small amount of clear colourless oil (8.6 mg, 0.03 mmol, 12%).

^{1}H NMR (400 MHz, CD_2Cl_2): δ 7.46 (d, 2H, ^{3}J(H,H) = 8.6 Hz, para-substituted benzene), 7.41 (d, 2H, ^{3}J(H,H) = 8.6 Hz, para-substituted benzene), 7.21 (dd, 2H, ^{3}J(H,H) =8.3), 7.08 (dd, 2H, ^{3}J(H,H) =8.3) ppm;
^{11}B NMR (128.4 MHz, CD_2Cl_2): δ 24.9 (s) ppm.

(Z)-2-(2-chloro-3,3-dimethylbut-1-en-1-yl)-4,4,5,5-tetramethyl-1,3,2-dioxaborolane

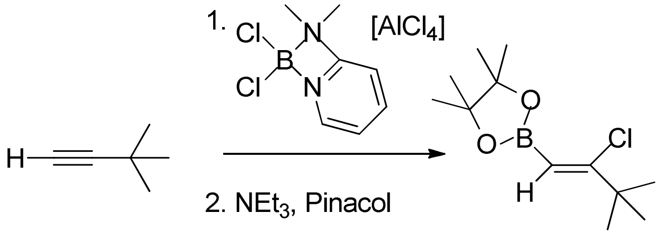

To a suspension of $[Cl_2B(2DMAP)][AlCl_4]$ (50 mg, 0.14 mmol) in anhydrous CH_2Cl_2 in a J.Young's NMR tube, ^{t}Bu acetylene (17 µl, 0.14 mmol) was added, turning light yellow and dissolving some of the precipitate. The reaction mixture was then stirred at room temperature for 18 hours, during which time the precipitate fully dissolved. NMR spectroscopy was used to confirm reaction completion, and the solution was esterified with excess triethylamine and 2 equivalents of pinacol. The solvent was removed under reduced pressure, leaving a yellow oil. Pentane was used to extract the product, which was passed through a 1 inch plug of silica to remove pinacol impurities. The product was isolated as a white oil (20 mg, 0.08 mmol, 63%).

^{1}H NMR (400 MHz, $CDCl_3$): δ 5.47 (s, 1H, vinyl), 1.24 (s, 12H, pinacol), 1.12 (s, 9H, ^{t}butyl) ppm;
^{13}C NMR (100.6 MHz, $CDCl_3$): δ 162.35, 83.56, 40.71, 28.81, 24.86, 24.80 ppm;
^{11}B NMR (128.4 MHz, $CDCl_3$): δ 29.8 (s) ppm.
Elemental Analysis
Calculated: C 62.52; H 9.07.
Observed: C 62.49; H 9.11.

Initial Product **5** prior to esterification**:**

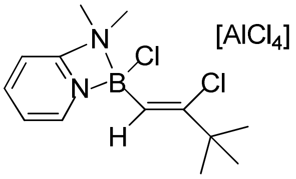

^{1}H NMR (400 MHz, $CDCl_3$): δ 8.77 (t, 1H), 8.67 (d, 1H), 8.26 (d, 1H), 8.15 (t, 1H), 5.81 (s, 1H, vinyl), 3.36 (s, 6H) ppm;
^{11}B NMR (128.4 MHz, $CDCl_3$): δ 12.2 (s) ppm.

(Z)-2-(2-chloro-2-phenylvinyl)-4,4,5,5-tetramethyl-1,3,2-dioxaborolane

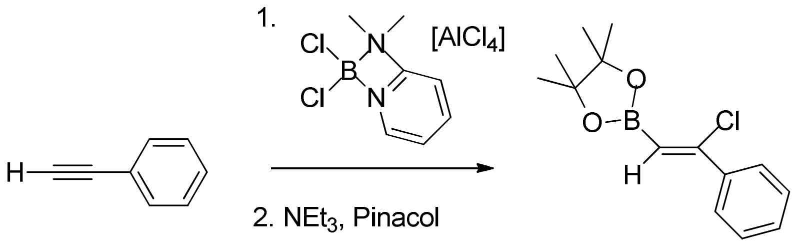

To a suspension of $[Cl_2B(2DMAP)][AlCl_4]$ (50 mg, 0.14 mmol) in anhydrous CH_2Cl_2 in a J.Young's NMR tube, phenylacetylene (15 µl, 0.14 mmol) was added, turning light yellow and dissolving some of the precipitate. The reaction mixture was then stirred at room temperature for 18 hours, during which time the precipitate fully dissolved. NMR spectroscopy was used to confirm reaction completion, and the solution was esterified with excess triethylamine and 2 equivalents of pinacol. The solvent was removed under reduced pressure, leaving a yellow oil. Pentane was used to extract the product, which was passed through a 1 inch plug of silica to remove pinacol impurities. The product was isolated as a yellow oil (30 mg, 0.12 mmol, 88%).

^{1}H NMR (400 MHz, $CDCl_3$): δ 7.61-7.58 (m, 2H, phenyl), 7.29-7.28 (m, 3H, phenyl), 6.04 (s, 1H, vinylic), 1.27 (s, 12H, pinacol) ppm;
^{13}C NMR (100.6 MHz, $CDCl_3$): δ 149.06, 138.99, 129.46, 128.26, 126.82, 83.74, 24.84 ppm;
^{11}B NMR (128.4 MHz, $CDCl_3$): δ 29.7 (s) ppm.
Elemental Analysis
Calculated: C 63.56; H 6.86.
Observed: C 63.52; H 6.90

(Z)-2-(2-chloropent-1-en-1-yl)-4,4,5,5-tetramethyl-1,3,2-dioxaborolane

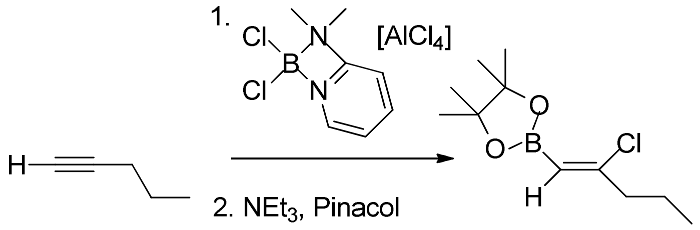

To a suspension of $[Cl_2B(2DMAP)][AlCl_4]$ (50 mg, 0.14 mmol) in anhydrous CH_2Cl_2 in a J.Young's NMR tube, 1-pentyne (17 µl, 0.14 mmol) was added, turning light yellow and dissolving some of the precipitate. The reaction mixture was then stirred at room temperature for 18 hours, during which time the precipitate fully dissolved. NMR spectroscopy was used to confirm reaction completion, and the solution was esterified with excess triethylamine and 2 equivalents of pinacol. The solvent was removed under reduced pressure, leaving a yellow oil. Pentane was used to extract the product, which was passed through a 1 inch plug of silica to remove pinacol impurities. The product was isolated as a white oil (26 mg, 0.11 mmol, 73%).

^{1}H NMR (400 MHz, $CDCl_3$): δ 5.39 (s, 1H, vinyl), 2.31 (t, 2H, alkyl), 1.53 (sextet, 2H, alkyl), 1.23 (s, 12H, pinacol), 0.85 (t, 3H, alkyl) ppm;
^{13}C NMR (100.6 MHz, $CDCl_3$): δ 153.89, 83.64, 38.39, 24.54, 21.75, 13.08 ppm;
^{11}B NMR (128.4 MHz, $CDCl_3$): δ 29.5 (s) ppm.
Elemental Analysis
Calculated: C 57.31; H 8.75.
Observed: C 57.36; H 8.77

(Z)-2-(2-chloro-2-(p-tolyl)vinyl)-4,4,5,5-tetramethyl-1,3,2-dioxaborolane

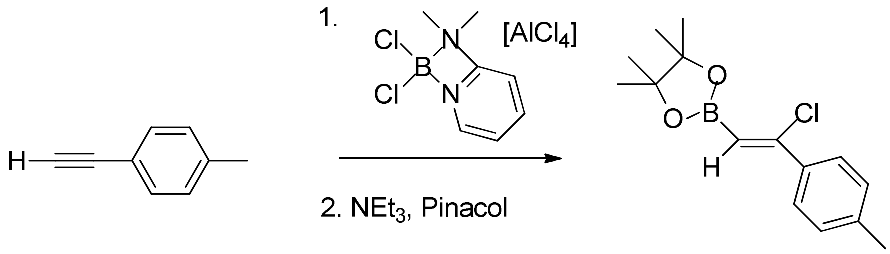

To a suspension of $[Cl_2B(2DMAP)][AlCl_4]$ (50 mg, 0.14 mmol) in anhydrous CH_2Cl_2 in a J.Young's NMR tube, 4-ethynyl toluene (17 µl, 0.14 mmol) was added, turning light yellow and dissolving some of the precipitate. The reaction mixture was then stirred at room temperature for 18 hours, during which time the precipitate fully dissolved. NMR spectroscopy was used to confirm reaction completion, and the solution was esterified with excess triethylamine and 2 equivalents of pinacol. The solvent was removed under reduced pressure, leaving a yellow oil. Pentane was used to extract the product, which was passed through a 1 inch plug of silica to remove pinacol impurities. The product was isolated as a yellow oil (24 mg, 0.09 mmol, 65 %).

^{1}H NMR (400 MHz, $CDCl_3$): δ 7.49 (d, 2H, phenyl), 7.08 (d, 2H, phenyl), 2.28 (s, 3H, methyl), 1.24 (s, 12H, pinacol) ppm;
^{13}C NMR (100.6 MHz, $CDCl_3$): δ 152.53, 140.12, 131.89, 129.96, 84.77, 21.89 ppm;
^{11}B NMR (128.4 MHz, $CDCl_3$): δ 29.5 (s) ppm.
Elemental Analysis
Calculated: C 64.67; H 7.24.
Observed: C 64.72; H 7.30

(Z)-2-(2-(4-bromophenyl)-2-chlorovinyl)-4,4,5,5-tetramethyl-1,3,2-dioxaborolane

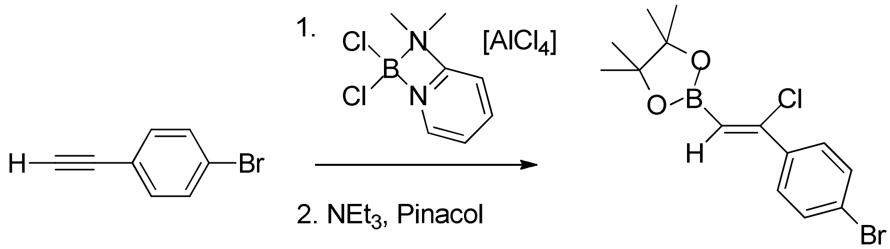

To a suspension of $[Cl_2B(2DMAP)][AlCl_4]$ (100 mg, 0.27 mmol) in anhydrous CH_2Cl_2 in a J.Young's NMR tube, 1-bromo-4-ethynylbenzene (49 µl, 0.27 mmol) was added, turning light yellow and dissolving some of the precipitate. The reaction mixture was then stirred at room temperature for 18 hours, during which time the precipitate fully dissolved. NMR spectroscopy was used to confirm reaction completion, and the solution was esterified with excess triethylamine and 2 equivalents of pinacol. The solvent was removed under reduced pressure, leaving a yellow oil. Pentane was used to extract the product, which was passed through a 1 inch plug of silica to remove pinacol impurities. The product was isolated as a yellow oil (63 mg, 0.18 mmol, 68%).
^{1}H NMR (400 MHz, $CDCl_3$): δ 7.38 (d, 2H, ^{3}J(H,H) = 8.6 Hz, para-substituted benzene), 7.31 (d, 2H, ^{3}J(H,H) = 8.6 Hz, para-substituted benzene), 5.61 (s, 1H, vinyl), 1.27 (s, 12H, pinacol) ppm;
^{13}C NMR (100.6 MHz, $CDCl_3$): δ 149.32, 139.59, 132.54, 129.89, 83.69, 24.93 ppm;
^{11}B NMR (128.4 MHz, $CDCl_3$): δ 29.3 (s) ppm.
Elemental Analysis
Calculated: C 48.96; H 4.98.
Observed: C 48.96; H 4.97

(Z)-2-(2-bromopent-1-en-1-yl)-4,4,5,5-tetramethyl-1,3,2-dioxaborolane

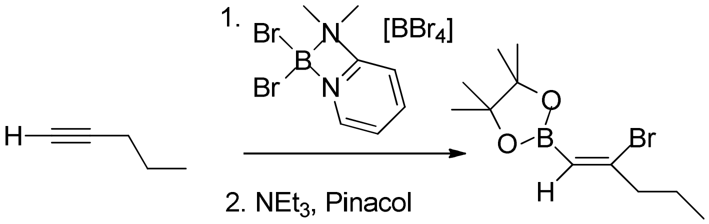

To a suspension of $[Br_2B(2DMAP)][BBr_4]$ (100 mg, 0.16 mmol) in anhydrous CH_2Cl_2 in a J.Young's NMR tube, 1-pentyne (16 µl, 0.16 mmol) was added, turning light yellow and dissolving some of the precipitate. The reaction mixture was then stirred at room temperature for 18 hours, during which time the precipitate fully dissolved. NMR spectroscopy was used to confirm reaction completion, and the solution was esterified with excess triethylamine and 2 equivalents of pinacol. The solvent was removed under reduced pressure, leaving a pale yellow oil. Pentane was used to extract the product, which was passed through a 1 inch plug of silica to remove pinacol impurities. The product was isolated as a yellow oil (34 mg, 0.12 mmol, 78%).

^{1}H NMR (400 MHz, $CDCl_3$): δ 5.80 (s, 1H, vinyl), 2.42 (t, 2H, alkyl), 1.54 (sextet, 2H, alkyl), 1.23 (s, 12H, pinacol), 0.85 (t, 3H, alkyl) ppm;
^{11}B NMR (128.4 MHz, $CDCl_3$): δ 29.4 (s) ppm.
Elemental Analysis
Calculated: C 48.04; H 7.33.
Observed: C 48.14; H 7.37

(E)-2-(4-bromohex-3-en-3-yl)-4,4,5,5-tetramethyl-1,3,2-dioxaborolane

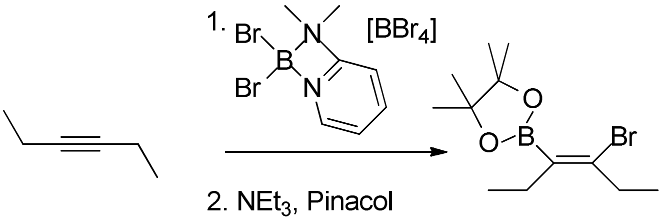

To a suspension of $[Br_2B(2DMAP)][BBr_4]$ (100 mg, 0.16 mmol) in anhydrous CH_2Cl_2 in a J.Young's NMR tube, 3-hexyne (19 µl, 0.16 mmol) was added, turning light yellow and dissolving some of the precipitate. The reaction mixture was then stirred at room temperature for 18 hours, during which time the precipitate fully dissolved. NMR spectroscopy was used to confirm reaction completion, as evidenced by desymeterisation of the 3-hexyne, and the solution was esterified with excess triethylamine and 2 equivalents of pinacol. The solvent was removed under reduced pressure, leaving a yellow oil. Pentane was used to extract the product, which was passed through a 1 inch plug of silica to remove pinacol impurities. The crude product was then purified using column chromatography in 1:1 DCM:petroleum ether. The product was isolated as a yellow oil (29 mg, 0.10 mmol, 62%).

^{1}H NMR (400 MHz, $CDCl_3$): δ 2.45 (q, 2H, ^{3}J(H,H) = 7.3 Hz), 2.10 (q, 2H, ^{3}J(H,H) = 7.6 Hz), 1.25 (s, 12H), 1.04 (t, 3H, ^{3}J(H,H) = 7.3 Hz), 0.94 (t, 3H, ^{3}J(H,H) = 7.7 Hz) ppm;
^{13}C NMR (100.6 MHz, $CDCl_3$): δ 134.43, 83.91, 30.73, 25.60, 24.78, 14.03, 13.24 ppm;
^{11}B NMR (128.4 MHz, $CDCl_3$): δ 30.2 (s) ppm.
Elemental Analysis
Calculated: C 49.69; H 7.65.
Observed: C 49.72; H 7.69

(E)-2-(3-bromo-4-methylpent-2-en-2-yl)-4,4,5,5-tetramethyl-1,3,2-dioxaborolane

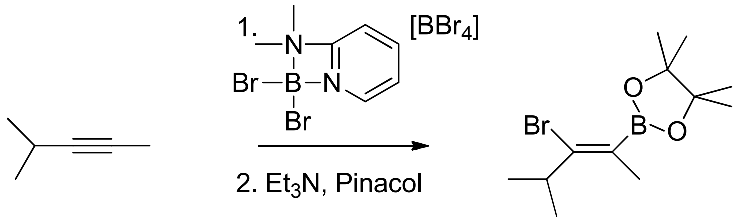

To a suspension of $[Br_2B(2DMAP)][BBr_4]$ (100 mg, 0.16 mmol) in anhydrous CH_2Cl_2 in a J.Young's NMR tube, 2-methyl-4-pentyne (0.16 mmol) was added, turning light yellow and dissolving some of the precipitate. The reaction mixture was then stirred at room temperature for 18 hours, during which time the precipitate fully dissolved. NMR spectroscopy was used to confirm reaction completion, as evidenced by desymeterisation of the 3-hexyne, and the solution was esterified with excess triethylamine and 2 equivalents of pinacol. The solvent was removed under reduced pressure, leaving a yellow oil. Pentane was used to extract the product, which was passed through a 1 inch plug of silica to remove pinacol impurities, and did not require further purification. The product was isolated as a colourless oil (24 mg, 0.08 mmol, 52 %).

^{1}H NMR (400 MHz, $CDCl_3$): δ 2.90 (septet, 1H,), 1.68 (s, 3H,), 1.25 (s, 12H), 0.96 (d, 6H,) ppm;
^{13}C NMR (100.6 MHz, $CDCl_3$): δ 83.88, 32.12, 24.69, 21.22, 17.12 ppm;
^{11}B NMR (128.4 MHz, $CDCl_3$): δ 30.1 (s) ppm.
Elemental Analysis
Calculated: C 49.87; H 7.67.
Observed: C 49.98; H 7.69.

nOe spectroscopy shows through space interaction between points 2.90 and both 1.68 and 0.96, but not with 1.25.

(Z)-2-(2-chloro-2-phenylvinyl)-4,4,5,5-tetramethyl-1,3,2-dioxaborolane

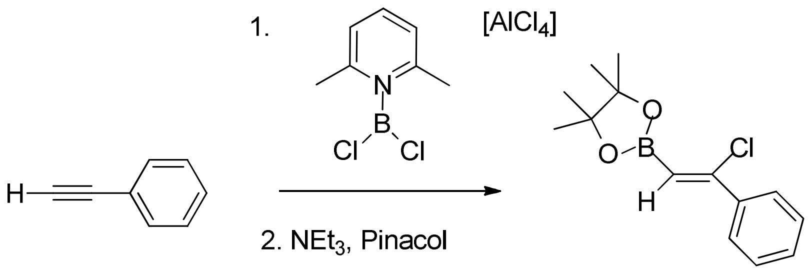

$LutBCl_3$ (50 mg, 0.22 mmol) was suspended in anhydrous CH_2Cl_2 in a J.Young's NMR tube, to which $AlCl_3$ (30 mg, 0.22 mmol) was added, causing dissolution to a clear yellow solution. To this $[LutBCl_2][AlCl_4]$, phenylacetylene (25 µl, 0.22 mmol) was added, turning dark brown. The reaction mixture was then stirred at room temperature for 4 hours, NMR spectroscopy confirmed reaction completion, and the solution was esterified with excess triethylamine and 2 equivalents of pinacol. The solvent was removed under reduced pressure, leaving an orange/brown oil. Pentane was used to extract the product, which was passed through a 1 inch plug of silica to remove pinacol impurities. The product was isolated as a yellow/orange oil (40 mg, 0.16 mmol, 71%).

^{1}H NMR (400 MHz, $CDCl_3$): δ 7.60-7.58 (m, 2H, phenyl), 7.29-7.27 (m, 3H, phenyl), 6.04 (s, 1H, vinylic), 1.27 (s, 12H, pinacol) ppm;
^{13}C NMR (100.6 MHz, $CDCl_3$): δ 149.07, 138.99, 129.47, 128.27, 126.82, 83.74, 24.85 ppm;
^{11}B NMR (128.4 MHz, $CDCl_3$): δ 29.6 (s) ppm.
Elemental Analysis
Calculated: C 63.56; H 6.86.
Observed: C 63.54; H 6.89.

Initial product

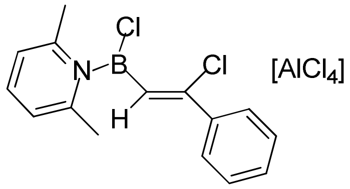

^{1}H NMR (400 MHz, $CDCl_3$): δ 8.45 (t, 1H), 7.92 (d, 2H), 7.86 (d, 2H), 7.64 (t, 2H), 7.53 (t, 2H), 7.23 (s, 1H, vinyl), 2.85 (s, 6H, lutidine) ppm;
^{11}B NMR (128.4 MHz, $CDCl_3$): δ 46.6 (br) ppm;
^{27}Al NMR (104 MHz, CD_2Cl_2): δ 103.4 (s) ppm.

(E)-2-(4-chlorohex-3-en-3-yl)-4,4,5,5-tetramethyl-1,3,2-dioxaborolane

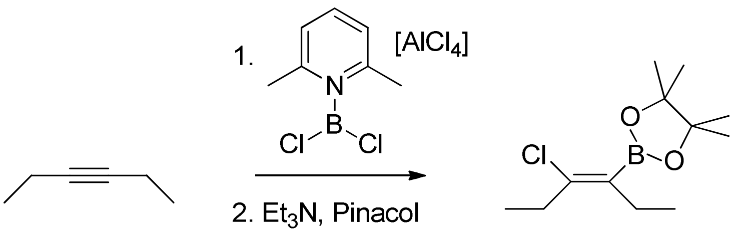

$LutBCl_3$ (50 mg, 0.22 mmol) was suspended in anhydrous *o*-$C_6H_4Cl_2$ in a J.Young's NMR tube, to which $AlCl_3$ (25 µl, 0.22 mmol) was added, causing dissolution to a clear yellow solution. To this $[LutBCl_2][AlCl_4]$, 3-hexyne (25 µl, 0.22 mmol) was added, turning dark brown. The reaction mixture was then stirred at room temperature for 24 hours, NMR spectroscopy confirmed reaction completion, and the solution was esterified with excess triethylamine and 2 equivalents of pinacol. The solvent was removed under reduced pressure, leaving an orange oil. Pentane was used to extract the product, which was passed through a 1 inch plug of silica to remove pinacol impurities. The product was isolated as a yellow/orange oil (44 mg, 0.18 mmol, 81 %).

^{1}H NMR (400 MHz, $CDCl_3$): δ 2.34 (q, 2H, ^3J(H,H) = 7.3 Hz), 2.10 (q, 2H, ^3J(H,H) = 7.6 Hz), 1.24 (s, 12H), 1.05 (t, 3H, ^3J(H,H) = 7.3 Hz), 0.93 (t, 3H, ^3J(H,H) = 7.7 Hz) ppm;
^{13}C NMR (100.6 MHz, $CDCl_3$): δ 142.69, 83.73, 28.63, 14.28, 12.54 ppm;
^{11}B NMR (128.4 MHz, $CDCl_3$): δ 30.3 (s) ppm.
Elemental Analysis
Calculated: C 58.93; H 9.07.
Observed: C 59.00; H 9.09
nOe spectroscopy shows through space interaction between points 1.05 and 0.93.

(E)-2-(1-chloro-1-phenylprop-1-en-2-yl)-4,4,5,5-tetramethyl-1,3,2-dioxaborolane

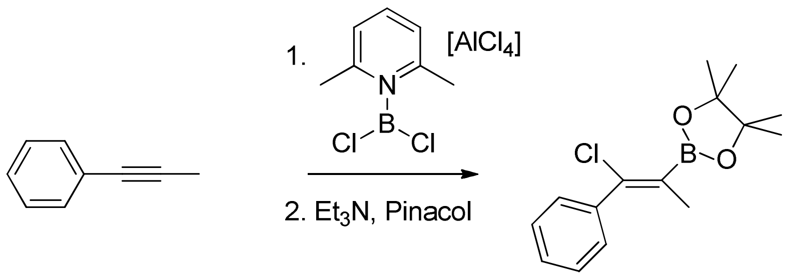

$LutBCl_3$ (50 mg, 0.22 mmol) was suspended in anhydrous *o*-$C_6H_4Cl_2$ in a J.Young's NMR tube, to which $AlCl_3$ (30 mg, 0.22 mmol) was added, causing dissolution to a clear yellow solution. To this [$LutBCl_2$][$AlCl_4$], 1-phenyl-1-propyne (28 µl, 0.22 mmol) was added, turning dark brown. The reaction mixture was then stirred at room temperature for 8 hours, NMR spectroscopy confirmed reaction completion, and the solution was esterified with excess triethylamine and 2 equivalents of pinacol. The solvent was removed under reduced pressure, leaving a yellow/orange oil. Pentane was used to extract the product, which was passed through a 1 inch plug of silica to remove pinacol impurities. The product was isolated as a yellow/orange oil (38 mg, 0.14 mmol, 62 %).

^{1}H NMR (400 MHz, $CDCl_3$): δ 7.27-7.23 (m, 3H, phenyl), 7.15-7.11 (m, 2H, phenyl), 2.06 (s, 3H, methyl), 1.22 (s, 12H, pinacol) ppm;
^{13}C NMR (100.6 MHz, $CDCl_3$): δ 132.58, 130.55, 128.37, 127.73, 24.63 ppm;
^{11}B NMR (128.4 MHz, $CDCl_3$): δ 30.4 (s) ppm.
Elemental Analysis
Calculated: C 64.67; H 7.18.
Observed: C 64.73; H 7.19
nOe spectroscopy shows through space interaction between points 7.23 (part of the multiplet corresponding to an *ortho* proton) and 2.06, and between 2.06 and 1.22.

(E)-2-(1-chloro-1-phenylbut-1-en-2-yl)-4,4,5,5-tetramethyl-1,3,2-dioxaborolane

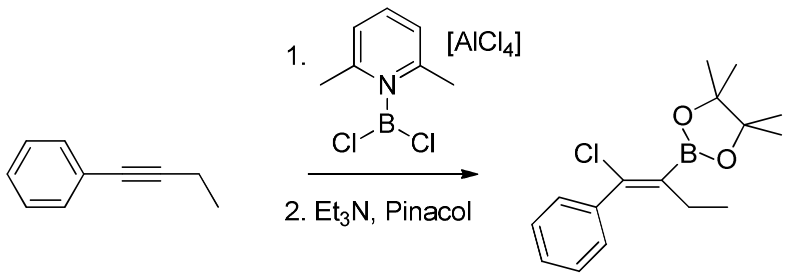

$LutBCl_3$ (50 mg, 0.22 mmol) was suspended in anhydrous *o*-$C_6H_4Cl_2$ in a J.Young's NMR tube, to which $AlCl_3$ (30 mg, 0.22 mmol) was added, causing dissolution to a clear yellow solution. To this $[LutBCl_2][AlCl_4]$, 1-phenyl-1-butyne (32 µl, 0.22 mmol) was added, turning dark brown. The reaction mixture was then stirred at room temperature for 18 hours, NMR spectroscopy confirmed reaction completion, and the solution was esterified with excess triethylamine and 2 equivalents of pinacol. The solvent was removed under reduced pressure, leaving an orange oil. Pentane was used to extract the product, which was passed through a 1 inch plug of silica to remove pinacol impurities. The product was isolated as a yellow oil (46 mg, 0.16 mmol, 72%).

^{1}**H NMR** (400 MHz, $CDCl_3$): δ 7.26-7.08 (m, 5H, phenyl), 2.31 (q, 2H, ^{3}J(H,H) = 7.4 Hz), 1.22 (s, 12H, pinacol), 1.06 (t, 3H, ^{3}J(H,H) = 7.4 Hz) ppm;
^{13}**C NMR** (100.6 MHz, $CDCl_3$): δ 144.52, 137.85, 129.50, 125.62, 83.08, 44.74, 28.28, 11.75 ppm;
^{11}**B NMR** (128.4 MHz, $CDCl_3$): δ 29.8 (s) ppm.
Elemental Analysis
Calculated: C 65.68; H 7.58.
Observed: C 65.6; H 7.57
nOe spectroscopy shows through space interaction between points 7.20 (part of the multiplet corresponding to an *ortho* proton) and 2.31, and between 1.22 and both 2.31 and 1.06.

(E)-2-(2-chloro-1,2-diphenylvinyl)-4,4,5,5-tetramethyl-1,3,2-dioxaborolane

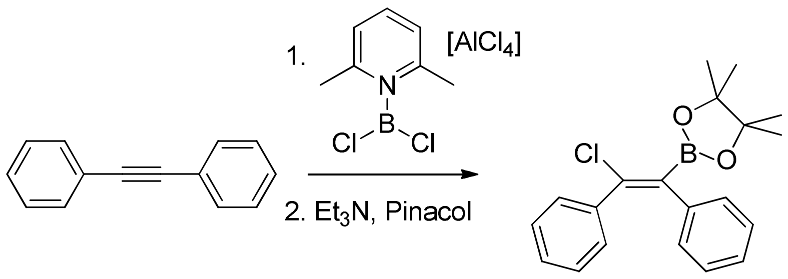

$LutBCl_3$ (50 mg, 0.22 mmol) was suspended in anhydrous *o*-$C_6H_4Cl_2$ in a J.Young's NMR tube, to which $AlCl_3$ (30 mg, 0.22 mmol) was added, causing dissolution to a clear yellow solution. To this [$LutBCl_2$][$AlCl_4$], diphenylacetylene (40 mg, 0.22 mmol) was added, turning dark brown. The reaction mixture was then stirred at room temperature for 24 hours, NMR spectroscopy confirmed reaction completion, and the solution was esterified with excess triethylamine and 2 equivalents of pinacol. The solvent was removed under reduced pressure, leaving a yellow/orange oil. Pentane was used to extract the product, which was passed through a 1 inch plug of silica to remove pinacol impurities. The product was isolated as a brown oil (32 mg, 0.09 mmol, 43 %).

^{1}H NMR (400 MHz, $CDCl_3$): δ 7.47-7.45 (m, 4H, phenyl), 7.29-7.24 (m, 6H, phenyl), 1.24 (s, 12H, pinacol) ppm;
^{11}B NMR (128.4 MHz, $CDCl_3$): δ 30.4 (s) ppm.
Elemental Analysis
Calculated: C 64.67; H 7.18.
Observed: C 64.73; H 7.19

(E)-2-(3-chloro-4-methylpent-2-en-2-yl)-4,4,5,5-tetramethyl-1,3,2-dioxaborolane

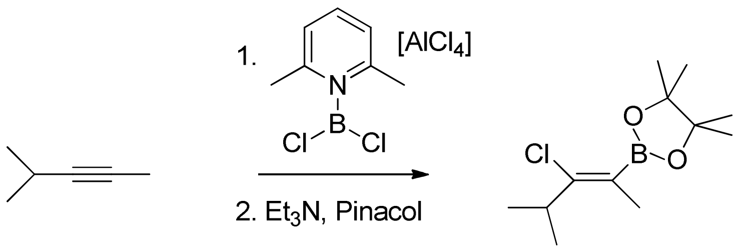

$LutBCl_3$ (50 mg, 0.22 mmol) was suspended in anhydrous *o*-$C_6H_4Cl_2$ in a J.Young's NMR tube, to which $AlCl_3$ (30 mg, 0.22 mmol) was added, causing dissolution to a clear yellow solution. To this $[LutBCl_2][AlCl_4]$, 2-methyl-4-pentyne (mg, 0.22 mmol) was added, turning dark brown. The reaction mixture was then stirred at room temperature for 18 hours, NMR spectroscopy confirmed reaction completion, and the solution was esterified with excess triethylamine and 2 equivalents of pinacol. The solvent was removed under reduced pressure, leaving a yellow/orange oil. Pentane was used to extract the product, which was passed through a 1 inch plug of silica to remove pinacol impurities. The product was isolated as a brown/orange oil (40 mg, 0.19 mmol, 83%).

^{1}H NMR (400 MHz, $CDCl_3$) δ 3.04 (septet, 1H, *i*-propyl), 1.69 (s, 3H, methyl), 1.24 (s, 12H), 0.99 (d, 6H, *i*-propyl) ppm;

^{13}C NMR (100.06 MHz, $CDCl_3$) δ 148.09, 83.72, 31.24, 24.69, 20.19, 16.07 ppm;

^{11}B NMR (128.4 MHz, $CDCl_3$): δ(s) 30.3 ppm.

Elemental Analysis

Calculated: C 58.93; H 9.07;

Observed: C 59.01; H 9.14;

nOe spectroscopy shows through space interactions between 1.69 and 3.04, 1.69 and 1.24 and 1.69 and 0.99.

(E)-2-(4-chloro-5-methylhexa-3,5-dien-3-yl)-4,4,5,5-tetramethyl-1,3,2-dioxaborolane

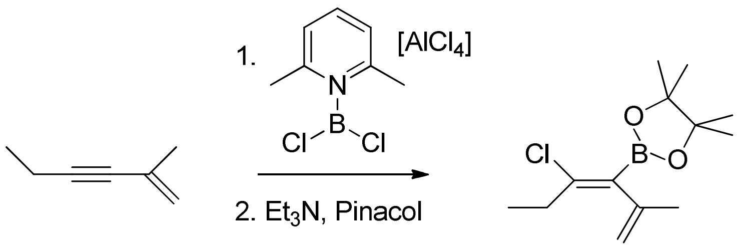

LutBCl$_3$ (50 mg, 0.22 mmol) was suspended in anhydrous *o*-$C_6H_4Cl_2$ in a J.Young's NMR tube, to which $AlCl_3$ (30 mg, 0.22 mmol) was added, causing dissolution to a clear yellow solution. To this [LutBCl$_2$][AlCl$_4$], 2-methylhexen-3-yne (28 µl, 0.22 mmol) was added, turning dark brown. The reaction mixture was then stirred at room temperature for 6 hours, NMR spectroscopy confirmed reaction completion, and the solution was esterified with excess triethylamine and 2 equivalents of pinacol. The solvent was removed under reduced pressure, leaving a yellow/orange oil. Pentane was used to extract the product, which was passed through a 1 inch plug of silica to remove pinacol impurities. The product was isolated as a light yellow oil (35 mg, 0.14 mmol, 61%).

^{1}H NMR (400 MHz, $CDCl_3$) δ 4.87 (m, 1H, vinyl), 4.64 (m, 1H, vinyl), 2.40 (q, 2H,ethyl), 1.76 (dd, 3H, methyl), 1.24 (s, 12H), 1.06 (t, 3H, ethyl) ppm;

^{13}C NMR (100.06 MHz, $CDCl_3$) δ 144.50, 143.53, 113.43, 83.96, 29.70, 24.64, 13.14 ppm;

^{11}B NMR (128.4 MHz, CDC13): δ29.9 (s) ppm.

Elemental Analysis

Calculated: C 60.85; H 8.64;

Observed: C60.89; H 8.67;

nOe spectroscopy shows through space interaction between points 4.64 and 2.40, also shows interaction between 1.24 and 1.76. Interaction between 4.87 and both 4.64 and 1.76 is observed.

(Z)-2-(2-chloro-2-(thiophen-2-yl)vinyl)-4,4,5,5-tetramethyl-1,3,2-dioxaborolane

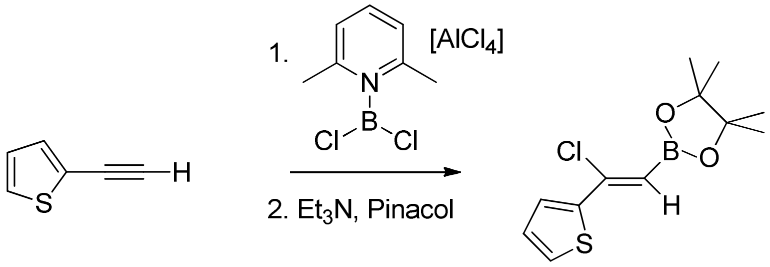

$LutBCl_3$ (50 mg, 0.22 mmol) was suspended in anhydrous *o*-$C_6H_4Cl_2$ in a J.Young's NMR tube, to which $AlCl_3$ (30 mg, 0.22 mmol) was added, causing dissolution to a clear yellow solution. To this [$LutBCl_2$][$AlCl_4$], diphenylacetylene (mg, 0.22 mmol) was added, turning dark brown. The reaction mixture was then stirred at room temperature for 18 hours, NMR spectroscopy confirmed reaction completion, and the solution was esterified with excess triethylamine and 2 equivalents of pinacol. The solvent was removed under reduced pressure, leaving a yellow/orange oil. Pentane was used to extract the product, which was passed through a 1 inch plug of silica to remove pinacol impurities. The product was isolated as a brown/orange oil (39 mg, 0.15 mmol, 65%).
^{1}H NMR (400 MHz, $CDCl_3$) δ 7.32 (dd, 1H,), 7.24 (dd, 1H,), 6.93 (dd, 1H), 5.99 (s, 1H, vinylic), 1.26 (s, 12H) ppm;
^{13}C NMR (100.06 MHz, $CDCl_3$) δ 141.7, 132.65, 130.87, 127.87, 127.72, 83.70, 24.83 ppm;
^{11}B NMR (128.4 MHz, $CDCl_3$): *δ* 29.5(s) ppm.
Elemental Analysis
Calculated: C 53.27; H 5.96;
Observed: C 53.24; H 6.00;
nOe spectroscopy shows through space interaction between points 7.24 and 5.99.

(E)-2-(1-bromo-2-chloro-2-phenylvinyl)-4,4,5,5-tetramethyl-1,3,2-dioxaborolane

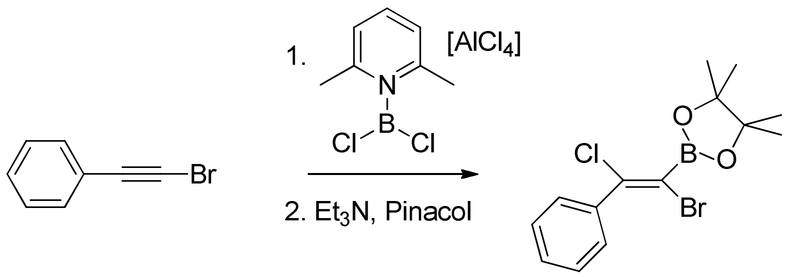

$LutBCl_3$ (50 mg, 0.22 mmol) was suspended in anhydrous *o*-$C_6H_4Cl_2$ in a J.Young's NMR tube, to which $AlCl_3$ (30 mg, 0.22 mmol) was added, causing dissolution to a clear yellow solution. To this [$LutBCl_2$][$AlCl_4$], diphenylacetylene (mg, 0.22 mmol) was added, turning dark brown. The reaction mixture was then stirred at room temperature for 18 hours, NMR spectroscopy confirmed reaction completion, and the solution was esterified with excess triethylamine and 2 equivalents of pinacol. The solvent was removed under reduced pressure, leaving a yellow/orange oil. Pentane was used to extract the product, which was passed through a 1 inch plug of silica to remove pinacol impurities. The product was isolated as a brown/orange oil (54 mg, 0.16 mmol, 71%).

^{1}H NMR (400 MHz, $CDCl_3$) δ 7.43-7.41 (m, 2H, phenyl), 7.32-7.31 (m, 3H, phenyl), 1.31 (s, 12H) ppm;
^{13}C NMR (100.06 MHz, $CDCl_3$) δ 138.64, 137.91, 129.37, 128.69, 128.18, 85.19, 24.55 ppm;
^{11}B NMR (128.4 MHz, $CDCl_3$): δ 28.4 (s) ppm.
Elemental Analysis
Calculated: C 48.96; H 4.99;
Observed: C 48.99; H 5.04;
(nOe shows no through space interaction between phenyl and pinacol resonances)

(Z)-2-(2-chloro-2-(4-methoxyphenyl)vinyl)-4,4,5,5-tetramethyl-1,3,2-dioxaborolane

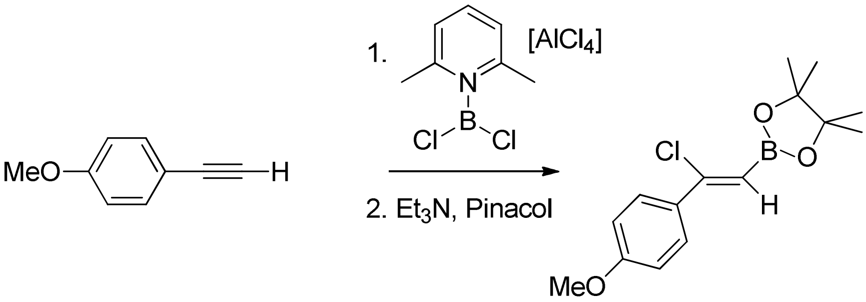

$LutBCl_3$ (50 mg, 0.22 mmol) was suspended in anhydrous *o*-$C_6H_4Cl_2$ in a J.Young's NMR tube, to which $AlCl_3$ (30 mg, 0.22 mmol) was added, causing dissolution to a clear yellow solution. To this $[LutBCl_2][AlCl_4]$, 4-ethynylanisole (30 mg, 0.22 mmol) was added, turning dark brown. The reaction mixture was then stirred at room temperature for 4 hours, NMR spectroscopy confirmed reaction completion, and the solution was esterified with excess triethylamine and 2 equivalents of pinacol. The solvent was removed under reduced pressure, leaving a yellow oil. Hexane was used to extract the product, which was passed through a 1 inch plug of silica to remove pinacol impurities. The product was isolated as a colourless oil (30 mg, 0.12 mmol, 52%).

^{1}H NMR (400 MHz, $CDCl_3$) δ 7.56 (d, 2H, phenyl), 6.80 (d, 2H, phenyl), 5.94 (s, 1H, vinylic), 3.76 (s, 3H, methoxy), 1.27 (s, 12H, pinacol) ppm;

^{13}C NMR (100.06 MHz, $CDCl_3$) δ 160.66, 148.94, 128.32, 113.55, 83.62, 55.13, 24.85 ppm;

^{11}B NMR (128.4 MHz, $CDCl_3$): δ30.4 (s) ppm.

Elemental Analysis

Calculated: C 61.16; H 6.84;

Observed: C 61.14; H 6.80;

(nOe shows through space interaction between the vinylic proton (5.94 ppm) and phenyl (7.56 ppm) resonance, but no interaction between phenyl and pinacol resonances)

(E)-2-(1-chloro-1-phenylpenta-1,4-dien-2-yl)-4,4,5,5-tetramethyl-1,3,2-dioxaborolane

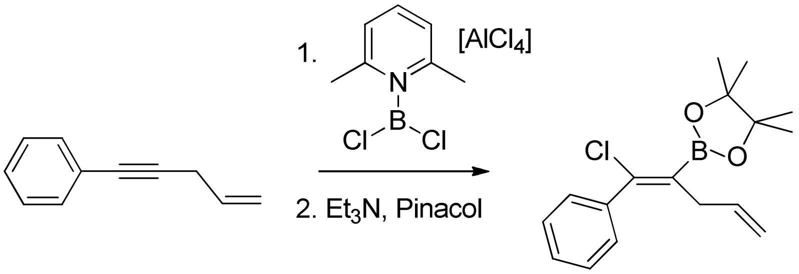

$LutBCl_3$ (50 mg, 0.22 mmol) was suspended in anhydrous *o*-$C_6H_4Cl_2$ in a J.Young's NMR tube, to which $AlCl_3$ (30 mg, 0.22 mmol) was added, causing dissolution to a clear yellow solution. To this $[LutBCl_2][AlCl_4]$, 1-phenyl-4-penten-1-yne (34 µl, 0.22 mmol) was added, turning dark brown. The reaction mixture was then stirred at room temperature for 4 hours, NMR spectroscopy confirmed reaction completion, and the solution was esterified with excess triethylamine and 2 equivalents of pinacol. The solvent was removed under reduced pressure, leaving a yellow/orange oil. Hexane was used to extract the product, which was passed through a 1 inch plug of silica to remove pinacol impurities. The product was isolated as a colourless oil (35 mg, 0.12 mmol, 53%).

^{1}H NMR (400 MHz, $CDCl_3$) δ 7.39-7.12 (m, 5H, phenyl), 5.76-5.69 (m, 1H, vinyl) 5.02-4.98 (dq, 1H, vinyl), 4.97-4.94 (dq, 1H, vinyl), 2.80 (dt, 2H, CH_2), 1.27 (s, 12H, pinacol) ppm;

^{13}C NMR (100.06 MHz, $CDCl_3$) δ 138.57, 138.04, 136.00, 128.40, 116.11, 84.14, 37.25, 24.77 ppm;

^{11}B NMR (128.4 MHz, $CDCl_3$): δ30.3 (s) ppm.

Elemental Analysis

Calculated: C 67.03; H 7.28;

Observed: C 67.00; H 7.27;

(nOe shows through space interaction between the CH_2 (2.81 ppm) and pinacol (1.27 ppm) resonances, but no interaction between pinacol and any phenyl resonances)

Reaction of **6** with PhCCH - Thermal stability test

A J. Young's NMR tube fitted with a d6-DMSO capillary was charged with Lutidine-BCl_3 adduct (40mg, 0.18mmol) and $AlCl_3$ (24mg, 0.18mmol) and DCM (1cm^3) added, the tube then gently inverted until dissolution was complete. Phenylacetylene (20µl, 0.18mmol) was added and a red colour formed immediately. Formation of the haloborated alkylborenium was quantitative. The reaction mixture was then refluxed at 60°C for 85 hours, after which the crude NMR spectra were found to be largely unchanged as seen in the example 1H NMR spectra below.

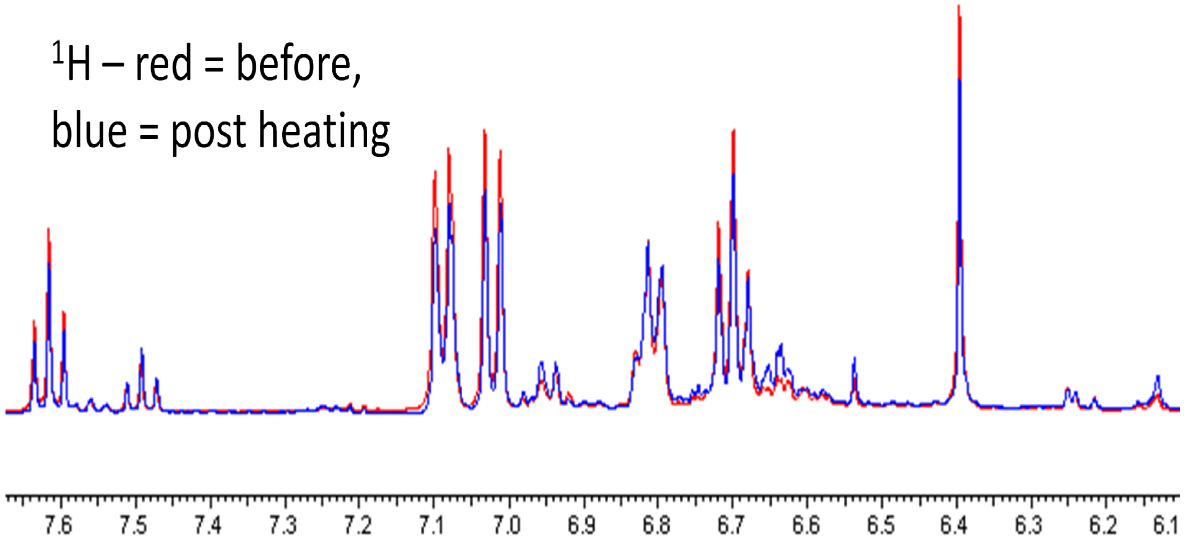

Reactivity of 1-pentyne with [BCl_2(2DMAP)][AlCl4] versus [BCl_2(2DMAP)][BArCl]

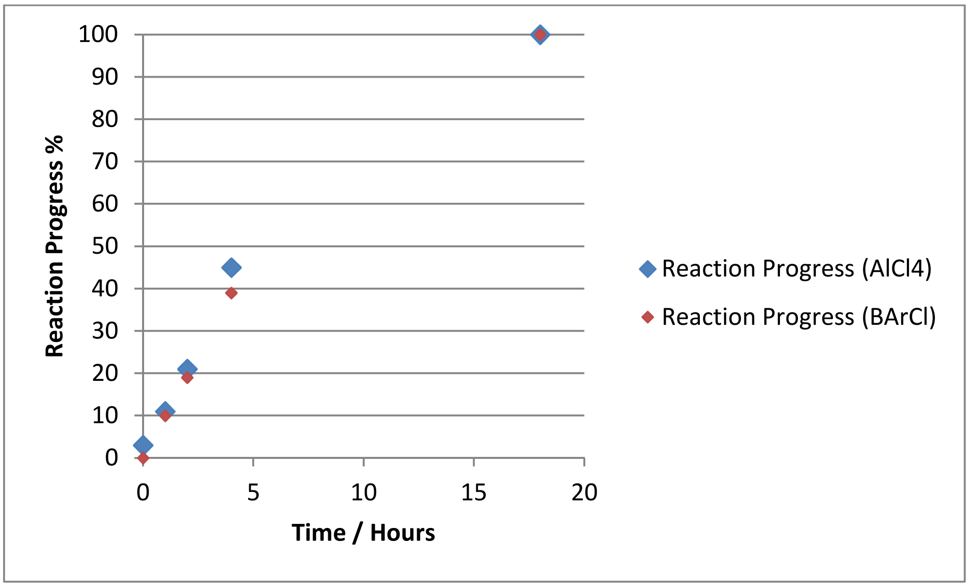

Reaction progress based on consumption of alkynyl C-H resonance and growth of vinylic C-H resonance by ^{1}H NMR spectroscopy (Vs. an internal standard), expressed as a percentage.

Cross Coupling Reactions

1-(1-chloro-1-phenylbut-1-en-2-yl)-4-methylbenzene

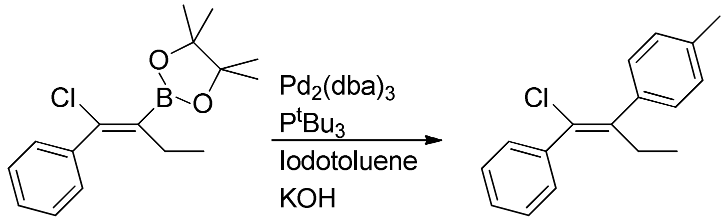

Under an inert atmosphere, $Pd_2(dba)_3$ (21 mg, 0.05eq), P^tBu_3 (18 mg, 0.2eq), alkene (E)-2-(1-chloro-1-phenylbut-1-en-2-yl)-4,4,5,5-tetramethyl-1,3,2-dioxaborolane (133 mg, 1eq) and 4-iodotoluene (99 mg, 1eq) were combined in THF (10 ml). To this, 3M KOH (454 µl, 3eq) was added and the reaction stirred for 18 hours. The reaction was quenched with aqueous ammonium chloride (10 ml) and extracted with ether (20 ml). The extract was washed with water, dried over $MgSO_4$, and concentrated under reduced pressure to yield a dark yellow oil. This crude product was purified with column chromatography using 3:1 hexane:DCM eluent. The product has an R_f value of 0.74, and presented as a light yellow oil (55 mg, 92 %)

^{1}H NMR (400 MHz, $CDCl_3$): δ 7.26 – 7.22 (m, 3H), 7.10 – 7.04 (m, 6H), 2.38 (q, 2H), 2.26 (s, 3H), 1.13 (t, 3H) ppm;

^{13}C NMR (100.6 MHz, $CDCl_3$): δ 137.96, 137.33, 136.86, 134.12, 128.47, 128.30, 128.01, 127.63, 127.27, 126.10, 123.16, 122.36, 29.27, 20.23, 12.11 ppm;

Successive Cross Coupling Reactions:

1-fluoro-4-(1-phenyl-2-(p-tolyl)but-1-en-1-yl)benzene

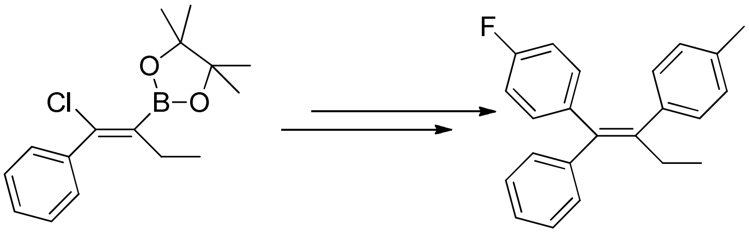

Under an inert atmosphere, $Pd_2(dba)_3$ (21 mg, 0.05eq), P^tBu_3 (18 mg, 0.2eq), alkene (E)-2-(1-chloro-1-phenylbut-1-en-2-yl)-4,4,5,5-tetramethyl-1,3,2-dioxaborolane (133 mg, 1eq) and 4-iodotoluene (99 mg, 1eq) were combined in THF (10 ml). To this, 3M KOH (454 µl, 3eq) was added and the reaction stirred for 18 hours. The reaction was quenched with aqueous ammonium chloride (10 ml) and extracted with ether (20 ml). The extract was washed with water, dried over $MgSO_4$, and concentrated under reduced pressure to yield a yellow oil, which was immediately used in the next step.

To the product, $Pd_2(dba)_3$ (24 mg, 0.05 eq), P^tBu_3 (21 mg, 0.2 eq), 4-fluorophenyl boronic acid (73 mg, 1eq) were added in THF (10 ml). To this, 3M KOH (518 µl, 3eq) was added and the reaction stirred for 18 hours. The reaction was quenched with aqueous ammonium chloride (10 ml) and extracted with ether (20 ml). The extract was washed with water, dried over $MgSO_4$, and concentrated under reduced pressure to yield a yellow/brown oil. This crude product was purified with column chromatography using 2:1 hexane:DCM eluent. The major product shows an R_f value of 0.60, and presented as a colourless oil (79 mg, 53 %)

^{1}H NMR (400 MHz, $CDCl_3$) δ 7.47-7.35 (m, 2H), 7.25-7.15 (m, 4H), 7.11-7.01 (m, 7H), 2.37 (q, 2H, alkyl CH_2), 2.25 (s, 3H, methyl), 1.12 (t, 3H, alkyl CH_3) ppm;
^{13}C NMR (100.06 MHz, $CDCl_3$) δ 140.14, 137.33, 136.86, 135.74, 134.11, 128.31, 128.00, 127.64, 127.27, 126.10, 114.76, 114.55, 29.28, 20.22, 12.10 ppm;
^{19}F NMR (376.50 MHz, $CDCl_3$) δ -115.70 ppm.

One Pot synthesis of 1-fluoro-4-(1-phenyl-2-(p-tolyl)but-1-en-1-yl)benzene

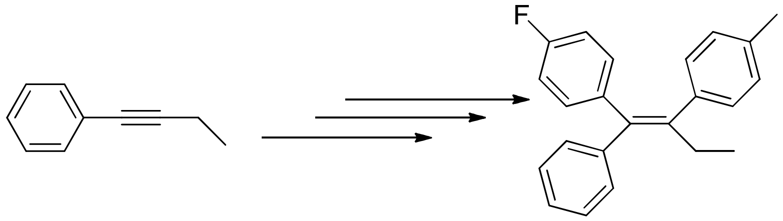

Under an inert atmosphere, 2,6-lutidine (1 eq) in hexane was added dropwise to boron trichloride (1M solution in heptanes, 1.2 eq) at 0˚C. This caused a precipitation of pale yellow-white solid; the Lut-BCl_3 adduct. After 20 minutes the solvent was removed under reduced pressure and the adduct suspended in dichlorobenzene. To this, aluminium trichloride was added to generate the desired borocation. After 20 minutes of stirring, the internal alkyne 1-phenyl-1-butyne (1 eq) was added dropwise, causing the suspention to go into solution, turning a yellow colour. The reaction was stirred for 18 hours, turning a dark orange, before transesterification with a solution of pinacol (2.1 eq) in excess triethylamine. Extraction with pentane and subsequent filtration through silica removed impurities to generate the pinacol boronate ester. (yield = 69%)

Under an inert atmosphere, $Pd_2(dba)_3$ (0.05 eq), P^tBu_3 (0.2eq), alkene (E)-2-(1-chloro-1-phenylbut-1-en-2-yl)-4,4,5,5-tetramethyl-1,3,2-dioxaborolane (1 eq) and 4-iodotoluene (1 eq) were combined in THF. To this, 3M KOH (3 eq) was added and the reaction stirred for 18 hours. The reaction was quenched with aqueous ammonium chloride and extracted with ether. The extract was washed with water, dried over $MgSO_4$, and concentrated under reduced pressure to yield a yellow oil, which was immediately used in the next step.

To the product, $Pd_2(dba)_3$ (0.05 eq), P^tBu_3 (0.2 eq), 4-fluorophenyl boronic acid (1 eq) were added in THF. To this, 3M KOH (3 eq) was added and the reaction stirred for 18 hours. The reaction was quenched with aqueous ammonium chloride and extracted with ether. The extract was washed with water, dried over $MgSO_4$, and concentrated under reduced pressure to yield a yellow/brown oil. This crude product was purified with column chromatography using 2:1 hexane:DCM eluent. The major product shows an R_f value of 0.60, and presented as a colourless oil (249 mg, 51 %)

^{1}H NMR (400 MHz, $CDCl_3$) δ 7.47-7.35 (m, 2H), 7.25-7.15 (m, 4H), 7.11-7.01 (m, 7H), 2.37 (q, 2H, alkyl CH_2), 2.25 (s, 3H, methyl), 1.12 (t, 3H, alkyl CH_3) ppm;
^{13}C NMR (100.06 MHz, $CDCl_3$) δ 140.14, 137.33, 136.86, 135.74, 134.11, 128.31, 128.00, 127.64, 127.27, 126.10, 114.76, 114.55, 29.28, 20.22, 12.10 ppm;
^{19}F NMR (376.50 MHz, $CDCl_3$) δ -115.70 ppm.

Minor Isomer (41 mg, 9 %)

^{1}H NMR (400 MHz, $CDCl_3$) δ 7.76 – 7.30 (m, 13H), 2.75 (q, 2H, alkyl CH_2), 2.56 (s, 3H, methyl), 1.49 (t, 3H, alkyl CH_3) ppm;
^{19}F NMR (376.50 MHz, $CDCl_3$) δ -116.27 ppm.

<u>X-ray Crystallography</u>

Data for compounds **1**, **4** and **5** were recorded on an Oxford Xcalibur Sapphire2 diffractometer, with Mo Kα radiation (graphite monochromator, λ=0.71073). The CrysAlisPro[2] software package was used for data collection, cell refinement and data reduction. Empirical absorption corrections were applied using spherical harmonics, implemented in SCALE3 ABSPACK scaling algorithm.

Data for compound **3** were recorded on a Bruker APEX-II diffractometer, with Cu Kα radiation (graphite monochromator, λ= 1.54178). The Bruker APEX2 software package was used for data collection, and the Bruker SAINT[3] software package was used for cell refinement and data reduction. Empirical absorption corrections were applied using SADABS-2008/1 - Bruker AXS area detector scaling and absorption correction

All structures were solved using direct methods[4] and refined against F^2 using the Crystals[5] software package. Non-hydrogen atoms were refined anisotropically. Hydrogen atoms were all located in a difference map and repositioned geometrically. Experimental details are given below in Table 1.

Table 1: Experimental details for X-ray structures of compounds, **1**, **3**, **4** and **5**

Name	Compound **1**	Compound **3**	Compound **4**	Compound **5**
Formula	$C_7H_{10}BCl_2N_2.AlCl_4$	$C_{13}H_{15}BClN_2.AlCl_4$	$C_{13}H_{14}BN_2O_2.AlCl_4$	$C_{13}H_{20}BCl_2N_2.AlCl_4$
CCDC Number	931221	931223	931220	931222
M_r	372.68	414.33	409.87	454.80
Crystal Size / mm	0.35 × 0.25 × 0.20	0.60 × 0.05 × 0.05	0.70 × 0.40 × 0.10	0.41 × 0.28 × 0.28
Crystal System	monoclinic	monoclinic	monoclinic	monoclinic
Space group	$P2_1/n$	$P2_1/c$	$P2_1/n$	$P2_1/n$
T / K	100	100	100	100
a /Å	12.3859(5)	9.8747(4)	9.9898(7)	13.6092(8)
b / Å	10.3851(4)	10.8803(5)	16.8931(18)	11.0861(5)
c / Å	12.6823(6)	17.5200(7)	10.4877(7)	15.0265(9)
β / °	101.692(4)	98.167(1)	91.183(6)	111.389(7)
V / Å^3	1597.47(12)	1863.25(14)	1769.5(3)	2110.9(2)
Z	4	4	4	4
$D_{calc.}$ / Mg.m^3	1.549	1.477	1.538	1.431
radiation	Mo *K*α, λ = 0.71073 Å	Cu *K*α, λ = 1.54178 Å	Mo *K*α, λ = 0.71073 Å	Mo *K*α, λ = 0.71073 Å
θ range (min-max) / °	3.2-28.4	4.5-72.8	3.0-28.6	2.9-27.4
reflns collected	10889	18506	7308	7335
indep reflns (R_{int})	3669 (0.051)	3658 (0.039)	3922 (0.056)	4073 (0.042)
reflns obsd I>2σ (I)	2870	3314	2824	2910
F(000)	744	840	832	928
R, wR for I>2σ (I)	0.043, 0.103	0.028, 0.071	0.052, 0.133	0.050, 0.054
R, wR for all data	0.063, 0.104	0.030, 0.071	0.084, 0.135	0.082, 0.084
S	1.03	1.00	1.02	1.03
$\Delta\rho_{max,\ min}$ / e·Å^{-3}	0.75, -0.70	0.40, -0.33	0.95, -0.98	0.39, -0.52

2) CrysAlisPro, Agilent Technologies, Version 1.171.35.19 (release 27-10-2011 CrysAlis171 .NET) (compiled Oct 27 2011,15:02:11)

3) APEX2 V2012.2-0

4) SIR92, Altomare, A., Cascarano, G., Giacovazzo, C., Guagliardi, A., Burla, M.C., Polidori, G. & Camalli, M. (1994). J. Appl. Cryst. 27, 435.

5) Crystals, Version 14.40b, January 2012, Betteridge, P.W., Carruthers, J.R., Cooper, R.I., Prout, K. & Watkin, D.J. (2003). J. Appl. Cryst. 36, 1487.

Computational Work
Calculations were performed using the Gaussian09 suite of programmes[6] Structures were pre-optimised at the HF/3-21G level followed by optimisation at the M06-2X/6-311G(d,p) level with inclusion of a PCM model for solvent correction (DCM)[7]. Transition states were located via the QST2 algorithm at the 3-21G level followed by optimisation at the M06-2X/6-311G(d,p) level with inclusion of a PCM model for solvent correction (DCM). In all cases, structures were confirmed as minima or transition states as appropriate by frequency analysis and the appropriate presence or absence of imaginary frequencies.

Full Cartesian coordinates for the M06-2X/6-311G(d,p) structures are provided below.

(6) Gaussian 09, Revision C1, Frisch, M. J.; Trucks, G. W.; Schlegel, H. B.; Scuseria, G. E.; Robb, M. A.; Cheeseman, J. R.; Scalmani, G.; Barone, V.; Mennucci, B.; Petersson, G. A.; Nakatsuji, H.; Caricato, M.; Li, X.; Hratchian, H. P.; Izmaylov, A. F.; Bloino, J.; Zheng, G.; Sonnenberg, J. L.; Hada, M.; Ehara, M.; Toyota, K.; Fukuda, R.; Hasegawa, J.; Ishida, M.; Nakajima, T.; Honda, Y.; Kitao, O.; Nakai, H.; Vreven, T.; Montgomery, Jr., J. A.; Peralta, J. E.; Ogliaro, F.; Bearpark, M.; Heyd, J. J.; Brothers, E.; Kudin, K. N.; Staroverov, V. N.; Kobayashi, R.; Normand, J.; Raghavachari, K.; Rendell, A.; Burant, J. C.; Iyengar, S. S.; Tomasi, J.; Cossi, M.; Rega, N.; Millam, J. M.; Klene, M.; Knox, J. E.; Cross, J. B.; Bakken, V.; Adamo, C.; Jaramillo, J.; Gomperts, R.; Stratmann, R. E.; Yazyev, O.; Austin, A. J.; Cammi, R.; Pomelli, C.; Ochterski, J. W.; Martin, R. L.; Morokuma, K.; Zakrzewski, V. G.; Voth, G. A.; Salvador, P.; Dannenberg, J. J.; Dapprich, S.; Daniels, A. D.; Farkas, Ö.; Foresman, J. B.; Ortiz, J. V.; Cioslowski, J.; Fox, D. J. Gaussian, Inc., Wallingford CT, 2009.

(7) http://comp.chem.umn.edu/info/DFT.htm]]

Summary of ring opening calculations:

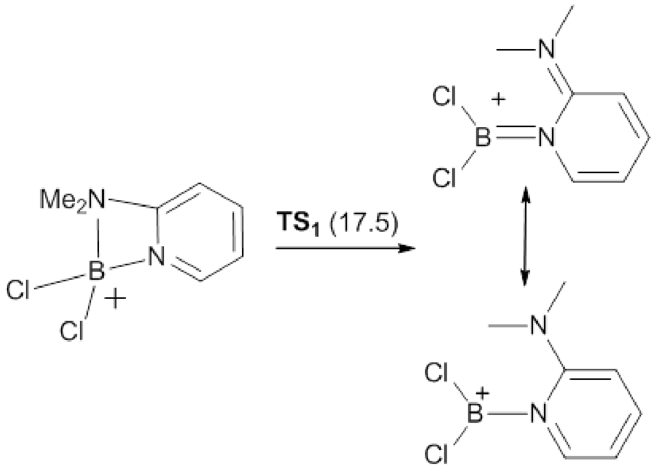

1 (0) **1_{RO} (12.3)**

Left, relative E (kcal mol^{-1}) in CH_2Cl_2 for **1**, **1_{RO}** and an intermediate transition state.

Boronium, **1**

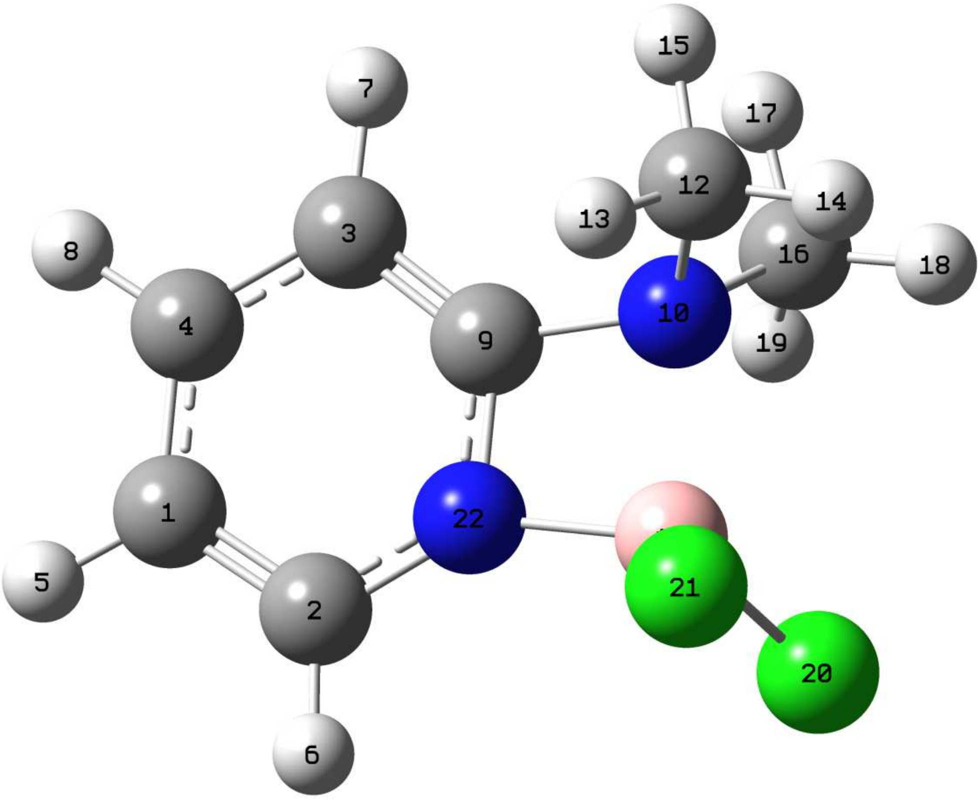

Center Number	Atomic Number	Coordinates (Angstroms) X	Y	Z
1	6	-2.850768	-1.292093	-0.019256
2	6	-1.515923	-1.663823	-0.008989
3	6	-2.226216	1.062651	0.009687
4	6	-3.198566	0.058613	-0.009077
5	1	-3.614692	-2.056395	-0.036013
6	1	-1.162223	-2.685724	-0.016052
7	1	-2.470765	2.115931	0.015683
8	1	-4.244601	0.337891	-0.017185
9	6	-0.935073	0.604048	0.017002
10	7	0.386184	1.206204	0.020547
11	5	0.944926	-0.391442	0.028264
12	6	0.669997	2.023172	1.240326
13	1	0.356123	1.476187	2.125178
14	1	1.743038	2.201023	1.275505
15	1	0.132433	2.966944	1.163038
16	6	0.657962	2.007393	-1.212669
17	1	0.069712	2.922701	-1.166981
18	1	1.721323	2.237430	-1.232292
19	1	0.386563	1.427053	-2.090213
20	17	1.808896	-0.866834	-1.472071
21	17	1.761546	-0.862040	1.557209
22	7	-0.605755	-0.687609	0.009000

Boronium 1 Ring Opening, TS1

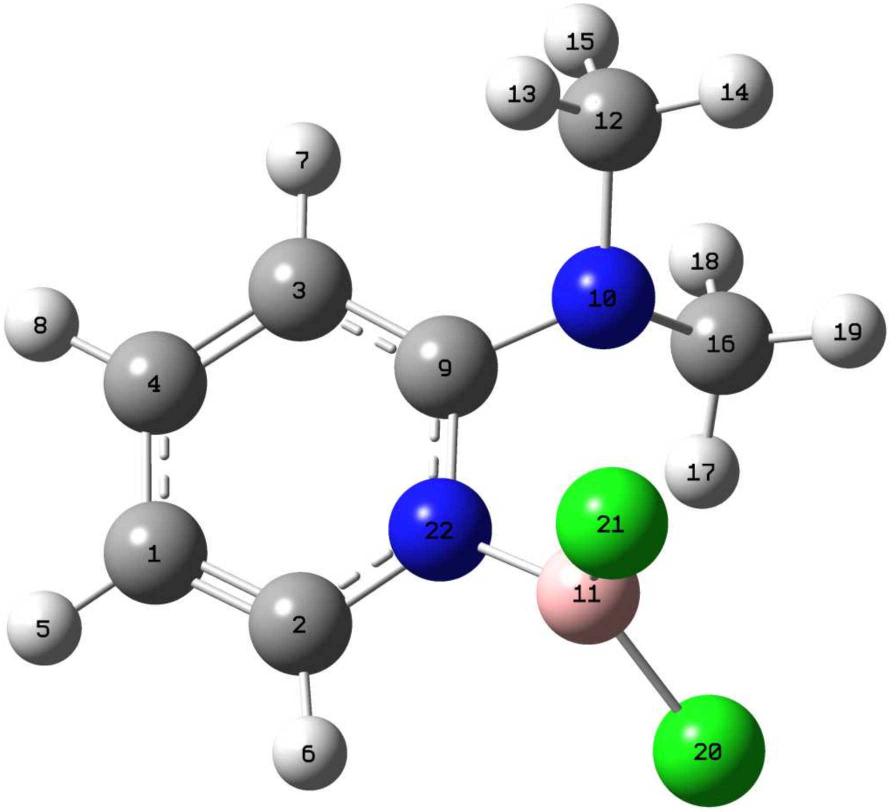

Center Number	Atomic Number	Coordinates (Angstroms) X	Y	Z
1	6	-2.023545	-2.215591	-0.067717
2	6	-0.679090	-1.967050	-0.169113
3	6	-2.417869	0.163347	0.106354
4	6	-2.896234	-1.135929	0.098884
5	1	-2.382515	-3.233737	-0.098751
6	1	0.065071	-2.747651	-0.261455
7	1	-3.084862	1.010184	0.187856
8	1	-3.960087	-1.314195	0.193495
9	6	-1.053448	0.378677	-0.047173
10	7	-0.396569	1.593206	-0.123024
11	5	1.251982	-0.410038	0.096139
12	6	-0.987687	2.695421	0.635715
13	1	-1.263560	2.350378	1.631193
14	1	-0.233178	3.475472	0.734000
15	1	-1.863720	3.118448	0.130938
16	6	0.035907	2.004160	-1.474796
17	1	0.336087	1.138407	-2.062890
18	1	-0.783045	2.513070	-1.993289
19	1	0.883835	2.681815	-1.379197
20	17	2.356668	-0.720397	-1.194271
21	17	1.780761	-0.005260	1.690258
22	7	-0.218730	-0.692182	-0.116934

Ring-open Borenium, $\mathbf{1_{RO}}$

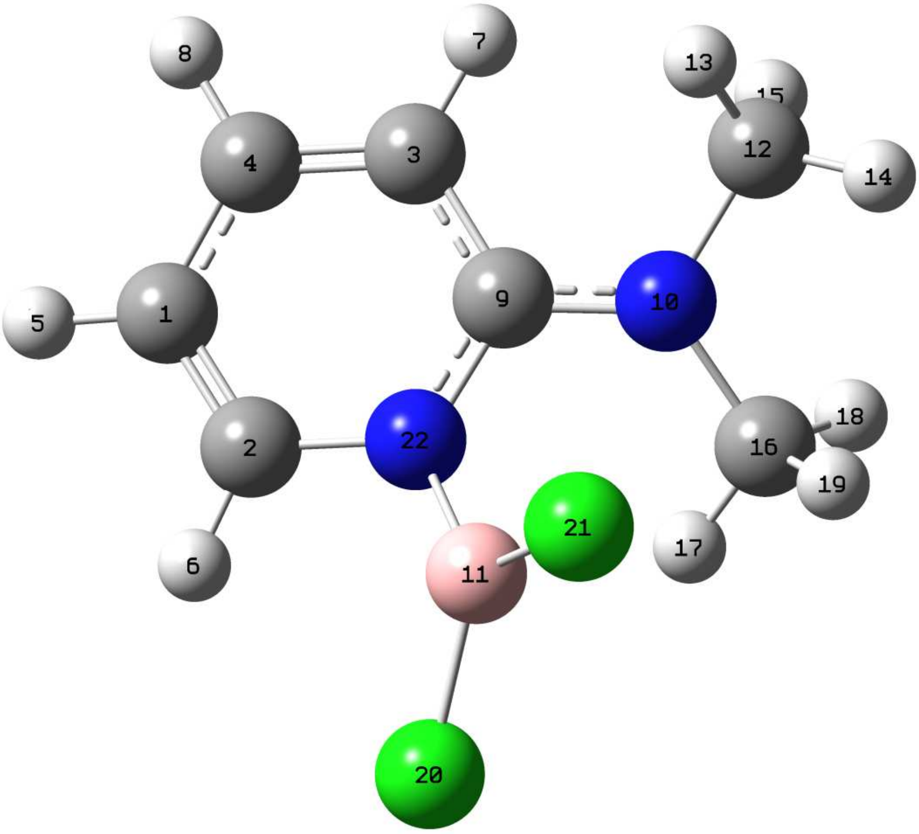

Center Number	Atomic Number	Coordinates (Angstroms) X	Y	Z
1	6	1.290041	2.661092	0.096319
2	6	0.108072	2.036794	-0.102913
3	6	2.381753	0.508030	0.209444
4	6	2.444458	1.867635	0.296213
5	1	1.331216	3.739633	0.117492
6	1	-0.824029	2.570336	-0.217721
7	1	3.277807	-0.087799	0.287922
8	1	3.399196	2.343531	0.482209
9	6	1.148446	-0.127337	-0.092546
10	7	1.063969	-1.425868	-0.375822
11	5	-1.342925	0.104035	0.156655
12	6	2.191831	-2.315129	-0.086847
13	1	2.618786	-2.082159	0.886205
14	1	1.809342	-3.333030	-0.057949
15	1	2.956242	-2.239471	-0.864421
16	6	0.036626	-2.013730	-1.236746
17	1	-0.583832	-1.240880	-1.690310
18	1	0.543050	-2.545451	-2.043624
19	1	-0.587108	-2.715017	-0.681762
20	17	-2.714685	0.752064	-0.683478
21	17	-1.548862	-1.079940	1.404792
22	7	0.009133	0.652369	-0.123680

Initial Association, **A**

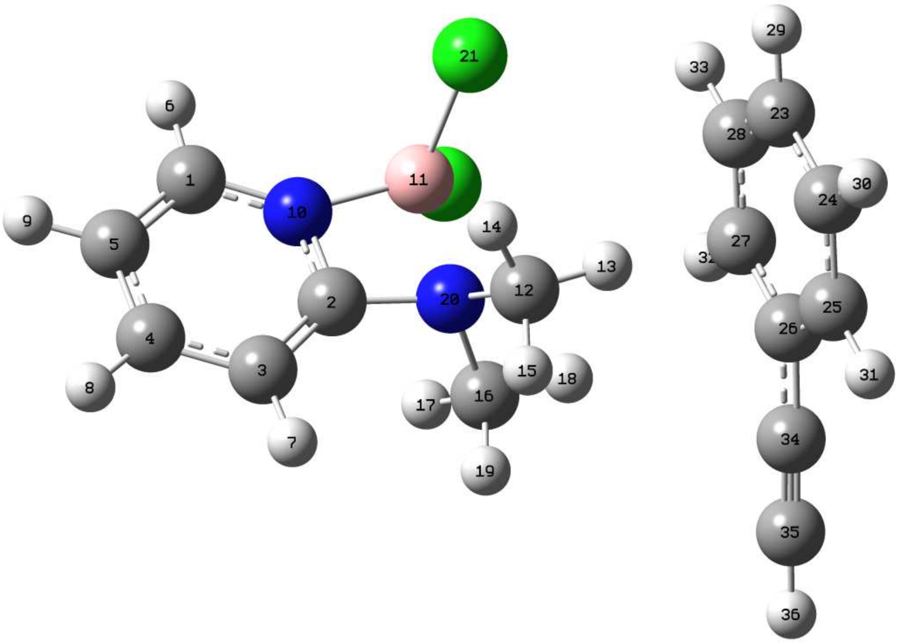

Center Number	Atomic Number	Coordinates (Angstroms) X	Y	Z
1	6	-0.684002	-0.354367	-2.220004
2	6	0.960601	0.423968	-0.746881
3	6	0.077016	0.947452	0.160435
4	6	-1.268924	0.783550	-0.180329
5	6	-1.649653	0.136317	-1.355821
6	1	-0.892464	-0.859593	-3.153275
7	1	0.394801	1.445572	1.065855
8	1	-2.031002	1.167745	0.485673
9	1	-2.694721	0.016937	-1.603417
10	7	0.593570	-0.187369	-1.872789
11	5	2.082209	-0.402200	-2.352392
12	6	3.122848	1.625045	-0.873806
13	1	4.138386	1.436751	-1.221768
14	1	2.617025	2.308057	-1.551374
15	1	3.135385	2.030165	0.136902
16	6	2.977948	-0.581297	0.196769
17	1	2.397365	-1.498878	0.244345
18	1	4.006708	-0.802986	-0.077532
19	1	2.945814	-0.060454	1.152929
20	7	2.404553	0.315077	-0.856916
21	17	2.507310	0.633932	-3.761737
22	17	2.612910	-2.112939	-2.476617
23	6	6.194125	1.298437	-3.169437
24	6	6.713412	2.000453	-2.084983
25	6	6.891284	1.366329	-0.860580
26	6	6.551074	0.016309	-0.716751
27	6	6.038078	-0.689558	-1.814146
28	6	5.859403	-0.047134	-3.032279
29	1	6.050433	1.796494	-4.120667
30	1	6.979105	3.045091	-2.191019
31	1	7.290550	1.907549	-0.011664
32	1	5.788505	-1.738689	-1.704423
33	1	5.457958	-0.597683	-3.874477
34	6	6.705565	-0.640468	0.549733
35	6	6.809689	-1.195099	1.611322
36	1	6.915704	-1.686367	2.550923

Vinyl Carbocation, **B**

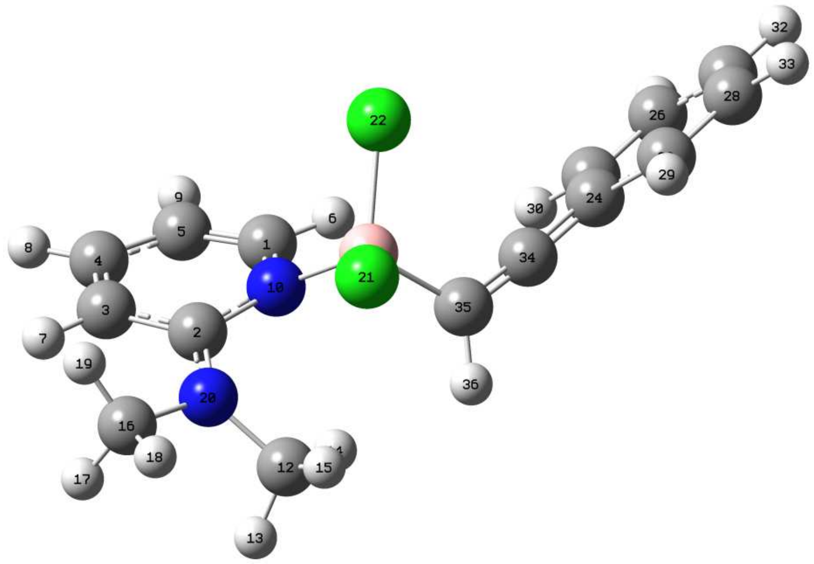

Center Number	Atomic Number	Coordinates (Angstroms) X	Y	Z
1	6	0.601177	-0.353336	-2.004982
2	6	2.326057	-1.596016	-3.033627
3	6	1.615260	-1.511724	-4.260102
4	6	0.464068	-0.779724	-4.332196
5	6	-0.022265	-0.116237	-3.187896
6	1	0.229149	0.058029	-1.076919
7	1	2.046508	-1.945905	-5.149371
8	1	-0.042799	-0.672020	-5.283200
9	1	-0.886130	0.530589	-3.224148
10	7	1.702855	-1.165914	-1.887573
11	5	1.847817	-1.813764	-0.438177
12	6	4.605169	-1.401254	-2.155410
13	1	5.325305	-0.909376	-2.815523
14	1	4.146903	-0.644071	-1.525943
15	1	5.121900	-2.133135	-1.536515
16	6	4.133722	-2.831176	-4.088228
17	1	4.462899	-2.195717	-4.917039
18	1	4.991979	-3.382642	-3.706062
19	1	3.396766	-3.550228	-4.440284
20	7	3.590610	-2.041510	-2.988803
21	17	2.948863	-3.276876	-0.337102
22	17	0.136913	-2.375314	0.096651
23	6	0.887763	-0.719693	3.797539
24	6	0.858580	0.055777	2.608954
25	6	-0.039734	1.145902	2.472031
26	6	-0.889451	1.446875	3.513068
27	6	-0.851813	0.674946	4.679754
28	6	0.028729	-0.401414	4.825957
29	1	1.580064	-1.548961	3.869978
30	1	-0.041573	1.720433	1.554063
31	1	-1.584234	2.271885	3.431699
32	1	-1.525172	0.917808	5.493390
33	1	0.031524	-0.980509	5.739495
34	6	1.696275	-0.276942	1.574420
35	6	2.418412	-0.658217	0.606384
36	1	3.426876	-0.269829	0.476190

Chloride Transfer TS, TS_{BC}

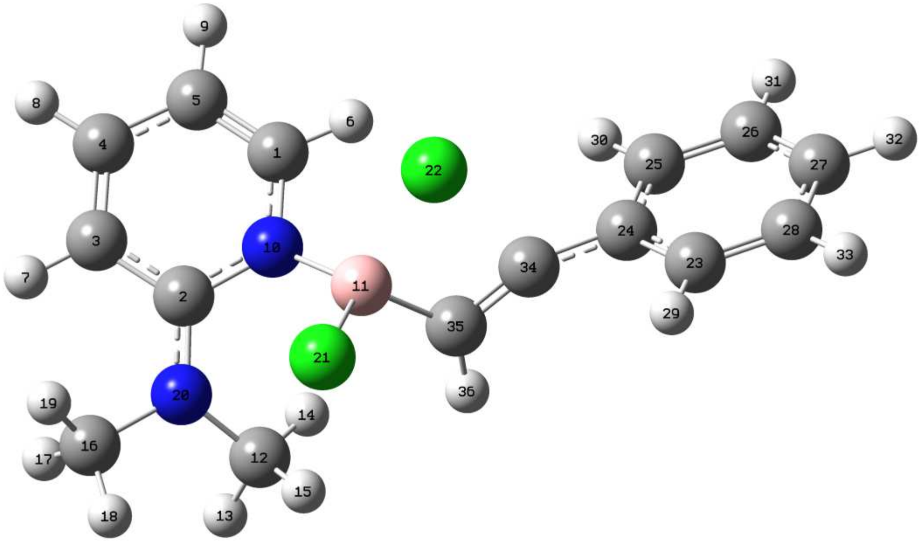

Center Number	Atomic Number	Coordinates (Angstroms) X	Y	Z
1	6	0.412502	-0.428347	-2.312524
2	6	2.364810	-1.501244	-3.107323
3	6	1.830508	-1.446431	-4.420113
4	6	0.637227	-0.821097	-4.643880
5	6	-0.060465	-0.231687	-3.570128
6	1	-0.108243	-0.047407	-1.447833
7	1	2.422700	-1.816738	-5.243202
8	1	0.256889	-0.736308	-5.654211
9	1	-0.966800	0.335416	-3.721179
10	7	1.556135	-1.147076	-2.052195
11	5	1.721901	-1.759804	-0.615909
12	6	4.443079	-1.237369	-1.833679
13	1	5.391835	-0.934499	-2.279207
14	1	3.945587	-0.341631	-1.467070
15	1	4.641402	-1.926685	-1.010477
16	6	4.380408	-2.627355	-3.873055
17	1	4.826186	-1.979437	-4.633787
18	1	5.174555	-3.159241	-3.350786
19	1	3.731716	-3.363850	-4.343226
20	7	3.636494	-1.854520	-2.882777
21	17	2.411146	-3.446422	-0.631982
22	17	-0.079069	-1.936198	0.224603
23	6	0.774760	-0.931374	3.756510
24	6	0.675741	-0.173781	2.575976
25	6	-0.020483	1.047431	2.555859
26	6	-0.595182	1.514981	3.723914
27	6	-0.486099	0.767712	4.897112
28	6	0.195648	-0.449105	4.916496
29	1	1.306598	-1.874704	3.742527
30	1	-0.090792	1.607568	1.631506
31	1	-1.128437	2.456608	3.727029
32	1	-0.939252	1.138577	5.808676
33	1	0.270023	-1.015328	5.835706
34	6	1.311261	-0.636636	1.402388
35	6	2.262779	-0.799636	0.540463
36	1	3.231828	-0.335576	0.699524

Vinyl Borocation, C

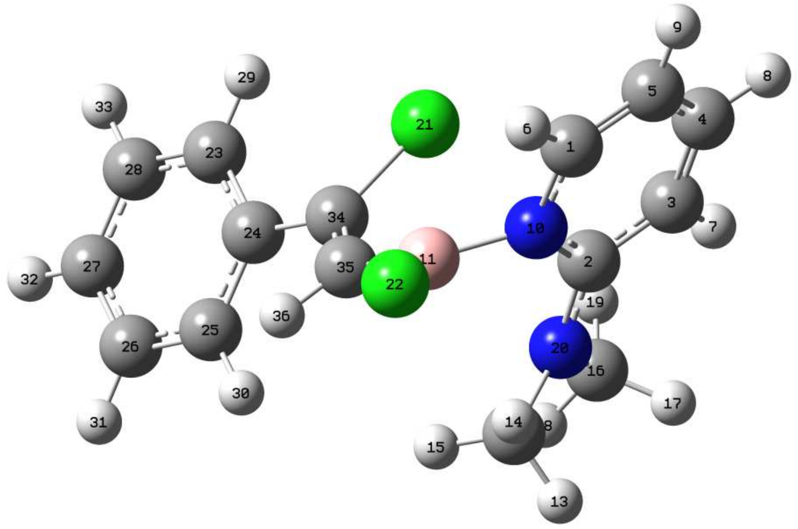

Center Number	Atomic Number	Coordinates (Angstroms) X	Y	Z
1	6	0.802007	-1.546066	1.111655
2	6	2.266025	-0.756075	-0.607001
3	6	2.109031	0.555005	-0.091472
4	6	1.327116	0.767750	1.007783
5	6	0.674223	-0.308709	1.648274
6	1	0.313016	-2.416080	1.526486
7	1	2.658681	1.367657	-0.541243
8	1	1.237993	1.768843	1.411089
9	1	0.081901	-0.165527	2.539495
10	7	1.536341	-1.770904	-0.035920
11	5	1.216131	-3.030369	-0.782706
12	6	3.792292	-2.309311	-1.777301
13	1	4.861720	-2.102593	-1.848159
14	1	3.637323	-2.946556	-0.906433
15	1	3.466918	-2.826184	-2.681150
16	6	3.605263	0.050128	-2.460097
17	1	4.460495	0.548029	-1.994649
18	1	3.920215	-0.387641	-3.405839
19	1	2.815466	0.771132	-2.663563
20	7	3.096993	-1.031785	-1.617624
21	17	-0.318021	-0.638057	-2.342391
22	17	1.277465	-4.521855	0.139552
23	6	-1.284195	-1.797140	-5.007202
24	6	-0.130471	-2.350888	-4.442993
25	6	0.730894	-3.108860	-5.245904
26	6	0.437138	-3.313150	-6.586374
27	6	-0.720243	-2.771085	-7.137961
28	6	-1.579675	-2.016170	-6.345708
29	1	-1.957193	-1.208698	-4.396601
30	1	1.643327	-3.516131	-4.827127
31	1	1.115500	-3.890250	-7.202344
32	1	-0.948381	-2.932938	-8.184373
33	1	-2.482231	-1.594678	-6.770277
34	6	0.197554	-2.166059	-3.016717
35	6	0.815438	-3.087439	-2.253772
36	1	0.975206	-4.045725	-2.741931

Ring Closing TS, TS_{CD}

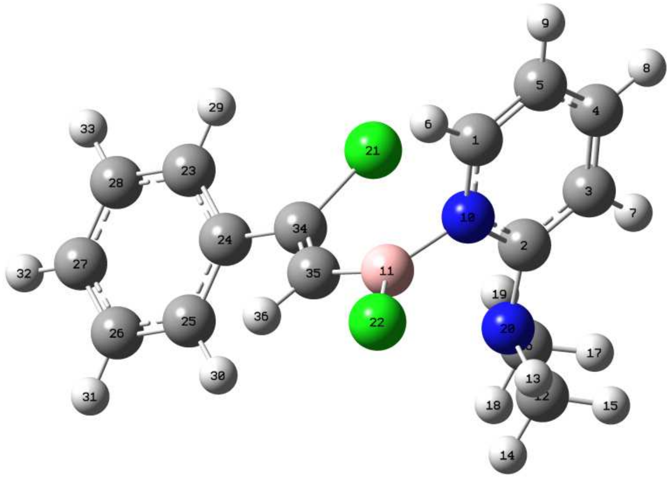

Center Number	Atomic Number	Coordinates (Angstroms) X	Y	Z
1	6	0.638332	-1.341903	0.615969
2	6	2.509899	-0.391192	-0.482389
3	6	2.269138	0.831036	0.131109
4	6	1.196614	0.943062	1.000653
5	6	0.382086	-0.160308	1.266914
6	1	0.034820	-2.232365	0.741469
7	1	2.931561	1.664056	-0.060351
8	1	1.005098	1.886629	1.496406
9	1	-0.446649	-0.098925	1.956987
10	7	1.663102	-1.425348	-0.262446
11	5	1.785640	-2.603126	-1.217605
12	6	4.684789	-1.401694	-0.731902
13	1	4.334033	-2.135661	-0.008107
14	1	5.246723	-1.914593	-1.511956
15	1	5.337635	-0.685499	-0.220841
16	6	3.974597	0.377426	-2.244565
17	1	4.596485	1.115760	-1.724711
18	1	4.563057	-0.064266	-3.048630
19	1	3.102408	0.866545	-2.677220
20	7	3.544015	-0.703876	-1.357485
21	17	0.150824	-0.143919	-2.617562
22	17	2.344921	-4.097601	-0.496115
23	6	-0.903921	-1.231773	-5.284522
24	6	0.283094	-1.775997	-4.782948
25	6	1.135428	-2.478128	-5.644152
26	6	0.798212	-2.640234	-6.980050
27	6	-0.393280	-2.112018	-7.468750
28	6	-1.242634	-1.410410	-6.618562
29	1	-1.568838	-0.684697	-4.628201
30	1	2.075519	-2.869447	-5.274225
31	1	1.470141	-3.172539	-7.641667
32	1	-0.655688	-2.241507	-8.511591
33	1	-2.171201	-0.999433	-6.994887
34	6	0.650168	-1.645276	-3.360791
35	6	1.311028	-2.588231	-2.661259
36	1	1.502448	-3.503803	-3.213806

Product Boronium, D

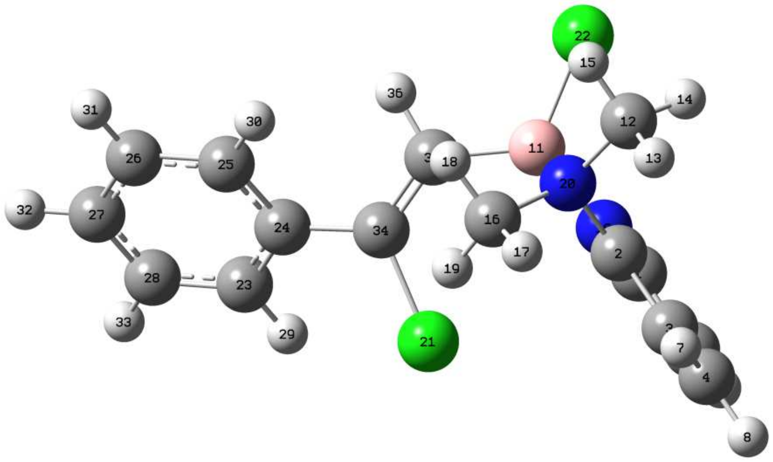

Center Number	Atomic Number	Coordinates (Angstroms) X	Y	Z
1	6	0.383837	-1.181096	0.364017
2	6	2.495468	-0.390649	-0.252306
3	6	2.417667	0.758218	0.492480
4	6	1.227353	0.908204	1.209341
5	6	0.219316	-0.054302	1.153134
6	1	-0.355046	-1.962601	0.250274
7	1	3.214747	1.488136	0.523316
8	1	1.087489	1.789556	1.822526
9	1	-0.695913	0.073847	1.713341
10	7	1.526259	-1.301136	-0.312371
11	5	2.267645	-2.179386	-1.398179
12	6	4.706739	-1.419351	-0.413558
13	1	5.282221	-0.536035	-0.138707
14	1	4.426078	-1.978537	0.474952
15	1	5.282894	-2.054592	-1.083522
16	6	3.830321	-0.146769	-2.316648
17	1	4.450593	0.687238	-1.990154
18	1	4.382394	-0.774461	-3.014080
19	1	2.923342	0.222512	-2.787601
20	7	3.474598	-0.988436	-1.138453
21	17	0.170949	0.010789	-2.735446
22	17	2.761086	-3.808942	-0.759859
23	6	-0.973800	-1.370967	-5.255346
24	6	0.342042	-1.659736	-4.883553
25	6	1.220704	-2.189819	-5.833713
26	6	0.784782	-2.441513	-7.127842
27	6	-0.531318	-2.167976	-7.487511
28	6	-1.407787	-1.632939	-6.548324
29	1	-1.660705	-0.952922	-4.529532
30	1	2.251785	-2.385147	-5.563569
31	1	1.476972	-2.843383	-7.857683
32	1	-0.870889	-2.364068	-8.497227
33	1	-2.433224	-1.416574	-6.822126
34	6	0.819195	-1.444760	-3.498508
35	6	1.672537	-2.242664	-2.851412
36	1	1.969273	-3.118993	-3.423944
